# Cross-national validation of the social media disorder scale: findings from adolescents from 44 countries

**DOI:** 10.1111/add.15709

**Published:** 2021-10-24

**Authors:** Maartje Boer, Regina J.J.M. van den Eijnden, Catrin Finkenauer, Meyran Boniel-Nissim, Claudia Marino, Jo Inchley, Alina Cosma, Leena Paakkari, Gonneke W.J.M. Stevens

**Affiliations:** 1 Department of Interdisciplinary Social Science, Utrecht University, Utrecht, the Netherlands; 2 Behavioural Sciences Department, Kinneret College on the Sea of Galilee, Jordan Valley, Israel; 3 Department of Developmental and Social Psychology, University of Padova, Padova, Italy; 4 MRC/CSO Social and Public Health Sciences Unit, University of Glasgow, Glasgow, UK; 5 Saints Cyril and Methodius Faculty of Theology, Olomouc University Social Health Institute, Palacký University Olomouc, Olomouc, Czechia; 6 Faculty of Electrical Engineering and Computer Science, VSB – Technical University of Ostrava, Ostrava, Czechia; 7 The Faculty of Sport and Health Sciences, University of Jyväskylä, Jyväskylä, Finland

**Keywords:** Adolescents, HBSC, international validation, problematic social media use, psychometric tests, social media addiction

## Abstract

**Background and aims:**

There is currently no cross-national validation of a scale that measures problematic social media use (SMU). The present study investigated and compared the psychometric properties of the social media disorder (SMD) scale among young adolescents from different countries.

**Design:**

Validation study.

**Setting and participants:**

Data came from 222 532 adolescents from 44 countries participating in the health behaviour in school-aged children (HBSC) survey (2017/2018). The HBSC survey was conducted in the European region and Canada. Participants were on average aged 13.54 years (standard deviation = 1.63) and 51.24% were girls.

**Measurement:**

Problematic SMU was measured using the nine-item SMD scale with dichotomous response options.

**Findings:**

Confirmatory factor analyses (CFA) showed good model fit for a one-factor model across all countries (minimum comparative fit index (CFI) and Tucker–Lewis index (TLI) = 0.963 and 0.951, maximum root mean square error of approximation (RMSEA) and standardized root mean square residual (SRMR) = 0.057 and 0.060), confirming structural validity. The internal consistency of the items was adequate in all countries (minimum alpha = 0.840), indicating that the scale provides reliable scores. Multi-group CFA showed that the factor structure was measurement invariant across countries (ΔCFI = -0.010, ΔRMSEA = 0.003), suggesting that adolescents’ level of problematic SMU can be reliably compared cross-nationally. In all countries, gender and socio-economic invariance was established, and age invariance was found in 43 of 44 countries. In line with prior research, in almost all countries, problematic SMU related to poorer mental wellbeing (range *β*_STDY_ = 0.193–0.924, *P* < 0.05) and higher intensity of online communication (range *β*_STDY_ = 0.163–0.635, P < 0.05), confirming appropriate criterion validity.

**Conclusions:**

The social media disorder scale appears to be suitable for measuring and comparing problematic social media use among young adolescents across many national contexts.

## Introduction

Adolescents are the most digitally connected age group world-wide [1]. Research among European adolescents shows that between 2017 and 2019, 77% of 15- and 16-year-olds reported daily use of social media [2], for instance Instagram and Snapchat. However, concerns have been raised about adolescents who display symptoms of addiction regarding social media use (SMU) [3], such as being unable to control SMU or by displacing other activities such as hobbies and sports for SMU [4,5]. However, diagnostic manuals including the Diagnostic and Statistical Manual of Mental Disorders (DSM-5) do not acknowledge social media addiction. Therefore, we refer to addiction-like SMU as ‘problematic SMU’. Cross-national data from the present Health Behaviour in School-aged Children (HBSC) study shows that, in 2017 and 2018, 4–18% of 15-year-olds reported problematic SMU [6].

With an increasing body of evidence suggesting that problematic SMU threatens different aspects of adolescents’ welbeing [7–10], different scales that measure problematic SMU have been developed. One of the most widely adopted scales is the Bergen Social Media Addiction Scale [11], which covers six items that parallel the core criteria of addiction, including preoccupation (i.e. salience), tolerance, withdrawal, persistence (i.e. relapse), escape (i.e. mood modification) and conflict [4,12]. However, this conceptualization may not sufficiently measure the detrimental impact of this behaviour for daily life, which is considered one of the core aspects of addiction-like behaviours [13,14]. Another scale that measures problematic SMU is the nine-item social media disorder (SMD) scale [5,15]. This scale includes the six core criteria and two additional criteria that measure detrimental consequences due to SMU, namely problems in important life domains and displacement of activities. It also includes the criterion deception. Together, these nine criteria parallel the criteria for internet gaming disorder, as listed in the appendix of the DSM-5 [16,17]. By including three additional criteria as well as the six core criteria, the SMD scale measures problematic SMU in a way that corresponds more with the scholarly and clinical definition of behavioural addictions, thereby possibly advancing the measurement of problematic SMU.

To our knowledge, validation studies on problematic SMU scales remain limited to single-country data [18–25], including validation studies on the SMD scale [5,15,26,27]. Studies among Dutch secondary school adolescents showed that the SMD scale had a solid unidimensional factor structure and adequate internal consistency. Also, higher values on the scale were associated with higher levels of compulsive internet use, self-declared social media addiction and problems related to mental health, sleep and school functioning, confirming convergent and criterion validity [5,15]. Research among US adolescents aged 13–19 years showed that the scale scores provided good internal consistency and correlated strongly with scores on alternative problematic SMU scales [26]. A study among Turkish adolescents aged 14–18 years used an adapted version of the SMD scale with polytomous response scales and showed adequate internal consistency and structural validity for a unidimensional scale [27].

Although these single-country validation studies suggest that the SMD scale has appropriate psychometric properties across some national contexts, these studies used different analyses and sample characteristics were diverse (e.g. with respect to age and representativeness), limiting the comparability of their findings. Adolescents’ problematic SMU can only be compared cross-nationally if it is measured with the same scale, which has been shown to be reliable and valid using identical analyses on comparable national samples. Furthermore, to secure comparability, the measurement properties should be invariant across countries to confirm that adolescents from different countries interpret the questions of the scale in a similar manner [28,29]. Cross-national research on problematic SMU is important to identify countries with particularly high levels of problematic SMU and to inform preventive actions to address the possible detrimental outcomes of problematic SMU [7–10]. Furthermore, international validation of a problematic SMU scale is crucial for obtaining more robust global knowledge about problematic SMU and identifying the extent to which it imposes a risk to adolescents’ health world-wide.

In response to the lack of cross-national validation of problematic SMU scales, the present study aimed to investigate the psychometric properties of the SMD scale using nationally representative crossnational data from the HBSC study. We examined the structural validity, reliability, measurement invariance and criterion validity of the scale. Thereby, we aim to establish whether the scale is suitable to measure and compare adolescent problematic SMU within a broad international context.

## Methods

### Sample

The HBSC study is a cross-sectional study that has been conducted every 4 years since 1983 in collaboration with the World Health Organization (WHO) Regional Office for Europe. The study monitors the health (behaviours) of 11-, 13- and 15-year-olds. The present study uses the 2017/18 survey, which includes nationally representative data from 47 countries and regions from the European Region and Canada. More specifically, it includes data from 45 countries and two regional subsamples for Belgium (Flanders and Wallonia). For consistency, we refer to the subsamples as countries. To ensure semantic equivalence across different languages and cultural settings, questionnaires were translated following a standardized protocol [30]. National research teams translated the English questionnaire into their national language and back-translated it into English, after which these translations were verified and corrected by language experts from the HBSC network [30,31]. All countries strictly followed the sampling method and data collection procedures as prescribed by the HBSC international research protocol, which involved sampling via randomly selected schools and classes [30]. Surveys were administered in classroom settings during school hours using digital (45%) or paper-and-pencil (55%) self-completion. Respondents were informed that participation was voluntary and anonymous. Active informed consent was obtained from schools and participants. Depending on the country, passive or active informed consent was obtained from parents. Participating countries obtained ethical approval of the study procedures from their institutional ethics committee [30].

### Measures

#### Problematic SMU

Problematic SMU was assessed with the nine-item SMD scale [5]. The questions were introduced with: ‘We are interested in your experiences with social media. The term social media refers to social network sites (e.g. Facebook, [add other local examples]) and instant messengers (e.g. [insert local examples], WhatsApp, Snapchat, Facebook messenger)’. Subsequently, respondents were asked: ‘During the past year, have you…’, followed by, for example, ‘regularly found that you can’t think of anything else but the moment that you will be able to use social media again?’ (preoccupation), with answer options 1, yes and 0, no. All items can be found in the Supporting information, Table S1. For the criterion validity analyses the sumscore of the scale was dichotomized, whereby adolescents reporting six to nine present symptoms were defined as a problematic user (1, problematic user: six to nine symptoms, 0, non-problematic user: no to five symptoms) [15,32]. This definition is based on a latent class analysis on the nine items in a nationally representative sample of Dutch adolescents aged 12–16 which identified three subgroups of users, whereby adolescents in the subgroup with the highest levels of problematic SMU reported six or more symptoms [15].

#### Mental wellbeing

We assessed two indicators of mental wellbeing. Life satisfaction was measured using the Cantril ladder, where respondents rated their life on a scale ranging from 0, worst possible life to 10, best possible life [33]. This measure has shown good test-re-test reliability and (crossnational) convergent validity with other mental wellbeing measures [34–36]. Psychosomatic complaints were measured using the eight-item HBSC Symptom Checklist [37]. Respondents were asked how often in the past 6 months they had experienced, for example, feeling low (psychological complaint) or headache (somatic complaint), with answer options ranging from 1, about every day to 5, rarely or never.

A mean score was computed after scores were rescaled, such that high scores indicate high levels of psychosomatic complaints (range = 1–5). Validation studies on the eight-item measure have shown adequate test−re-test reliability, good content validity and high factor loadings (> 0.50) across different national settings [37,38].

#### Intensity of online communication

A newly developed four-item measure, adapted from the EU Kids Online Survey on the frequency of online communication with different contacts [39], was used. Respondents were asked how often they have online contact through social media with close friends, friends from a larger friend group, friends they met through the internet and other people (e.g. parents, siblings, classmates, teachers). Answer options ranged from 1, never/almost never to 5, almost all the time throughout the day, and a ‘do not know/does not apply’ option. The intensity of online communication was defined by the maximum score of the four items. Hence, higher scores indicate higher intensity of online communication (range = 1–5).

#### Demographic characteristics

Gender was assessed by asking respondents whether they are boy or girl (1, girl, 0, boy). Age was computed based on the respondent’s month and year of birth and the date of the survey assessment. For the measurement invariance analysis, respondents were assigned to three categories: 11- (≥ 10 and ≤ 12.5), 13- (> 12.5 and ≤ 14.5) and 15-year-olds (> 14.5 and ≤ 16.5). Socio-economic status was measured with the six-item Family Affluence Scale (FAS) [40], which assesses material assets in the household (e.g. number of cars). Sumscores were computed and transformed into proportional ranks given their residential country [41] and subsequently divided into three categories (1, lowest 20%, 2, middle 60% and 3, highest 20%).

### Analyses

#### Missing data

Missing data on the study variables were imputed based on multiple imputation with chained equations [42]. Five imputations were generated using predictive mean matching with five ‘nearest neighbours’ and logistic regression for the dichotomous items, predicted by the available data on the study measures, demographic characteristics, other wellbeing indicators and residential country to control for the nested structure of the data.

#### Structural validity

The structural validity defines the extent to which the scores on the scale reflect the underlying dimension. The SMD scale was developed as a unidimensional scale [5,15]. Hence, we evaluated the factor structure of the scale based on CFA of a one-factor model, based on the comparative fit index (CFI), Tucker Lewis index (TLI), root mean square error of approximation (RMSEA) and standardized root mean square residual (SRMR) (CFI/TLI = ≥ 0.90 acceptable, ≥ 0.95 good; RMSEA = ≤ 0.08 acceptable, ≤ 0.06 good; and SRMR = ≤ 0.10 acceptable, ≤ 0.08 good) [43]. We did not rely on the χ^2^ statistic, given its sensitivity to large sample sizes [44]. Solid structural validity was established when the model fit was acceptable and at least five items had factor loadings of 0.50 or higher [45]. Prior to the CFA, we conducted exploratory factor analysis (EFA) for each country to consolidate the proposition that the SMD scale measures one underlying dimension [5,15]. The EFA and CFA were conducted on different random subsamples, referring to calibration (EFA) and validation (CFA) subsamples.

#### Reliability

Reliability was assessed based on the internal consistency of the scores on the nine items using the validation subsamples. Given the dichotomous nature of the nine items, we computed the internal consistency using the tetrachoric correlation matrix, referred to as the ordinal alpha [46]. An alpha of 0.80 or higher indicates good reliability [46].

#### Measurement invariance

Measurement invariance means that the scale measures the same underlying construct across subpopulations, which is required in order to reliably compare the level of problematic SMU across subpopulations [28]. To do so, we examined whether the factor structure was comparable across countries (44 countries), gender (boy and girl), age groups (11-, 13- and 15-year-olds) and socio-economic status (low, middle and high family affluence) using multi-group CFA. We compared the model fit of a multi-group CFA where all item factor loadings and thresholds were free to vary across countries or subgroups (i.e. configural invariance), with the model fit of a multi-group CFA where all item factor loadings and thresholds were constrained to be equal across all countries or subgroups (i.e. scalar invariance) using the default model settings [47]. A test of loading invariance where thresholds are freely estimated (i.e. metric invariance) was not conducted because this model is not identified when using dichotomous items [47]. Measurement invariance was established when the scalar model decreased CFI by not more than 0.010 and increased RMSEA by not more than 0.015, relative to the configural model [48,49].

#### Criterion validity

Criterion validity refers to the extent to which a construct relates to another construct that it should theoretically be related to. Research suggests that problematic SMU impairs mental health [10,50], and that problematic users use online communication intensively [51,52]. Accordingly, review studies show a small to moderate negative association between problematic SMU and positive mental wellbeing, such as life satisfaction, and a positive moderate association between problematic SMU and negative mental wellbeing, for instance depression [7,53]. Review studies on problematic SMU and the frequency of or time spent on SMU (including activities such as browsing, chatting) show a small to moderate association [52,54], which may also apply to the relation between problematic SMU and online communication intensity. Hence, appropriate criterion validity would be established when problematic SMU was negatively related to life satisfaction with small to moderate effect size, positively to psychosomatic complaints with moderate effect size and positively to the intensity of online communication with small to moderate effect size (P < 0.05). Associations were examined using linear regression where problematic SMU predicted life satisfaction, psychosomatic complaints and online communication, while controlling for gender, age and socio-economic status. Estimates of problematic SMU were standardized to interpret their effect size. As the problematic SMU scores were dichotomous, estimates were STDY standardized (0.2 = small, 0.5 = moderate, 0.8 = large effect size) [55,56].

#### Technical details

Missing data were imputed using Stata version 13.0 [57]. Analyses were conducted on the imputed datasets with Mplus version 8.5 [58]. The CFAs, internal consistency and measurement invariance analyses were conducted using weighted least square means and variance adjusted (WLSMV) estimation with a probit regression link, as appropriate for analyses with categorical outcomes [59]. Regression analyses from the criterion validity analysis were conducted with maximum likelihood with robust standard errors (MLR). In all analyses, standard errors were corrected for clustering of adolescents within schools or classes. For some countries, the analyses were conducted using sample weights to adjust for sample distribution differences with the respective population. Analyses by country were conducted with the *MplusAutomation* package in RStudio 1.2.5042 [60,61]. All codes related to the analyses may be consulted via https://osf.io/bgkec/. The analyses were not pre-registered, and therefore results should be considered exploratory.

## Results

### Sample characteristics

The initial sample includes 47 countries (*n* = 244 097). Three countries were excluded because they did not survey problematic SMU (n = 10 576). Adolescents who responded ‘not applicable/don’t know’ to all items of the intensity of online communication scale automatically skipped the questions on problematic SMU and were also excluded from the sample (ranging from 1.78% in North Macedonia to 17.62% in Azerbaijan, n = 10 989). This yielded a sample of 222 532 adolescents from 44 countries (listed in the tables from the Supporting information). From these countries, the average school and participant response rates were 69.70 and 80.34%, respectively [62]. Adolescents were, on average, aged 13.54 years [standard deviation (SD) = 1.63, min. = 10.00, max. = 16.50] and 51.24% were girls.

Cronbach’s alpha for psychosomatic complaints was 0.81, which indicates good reliability [46]. Cronbach’s alpha was not calculated for the other study measures, because they either consisted of one item (life satisfaction) or were considered as a formative scale (intensity of online communication, socio-economic status), which means that not all items were expected to have high intercorrelations [63].

Missing data on the study measures ranged between 0.65 (age) and 10.14% (problematic SMU: escape). Little’s χ^2^ test for missing data showed that these data were not completely missing at random (χ^2^_(55 103)_ = 82498.58, *P* < 0.001), which implies that imputation of missing data is required in order to prevent potential bias [64].

### Prevalence differences

Table 1 shows that the most prevalent symptoms were ‘persistence’ (30.66%) and ‘escape’ (30.74%). The least prevalent symptoms were ‘conflict’ (14.38%) and ‘deception’ (14.56%).

**Figure 1:**
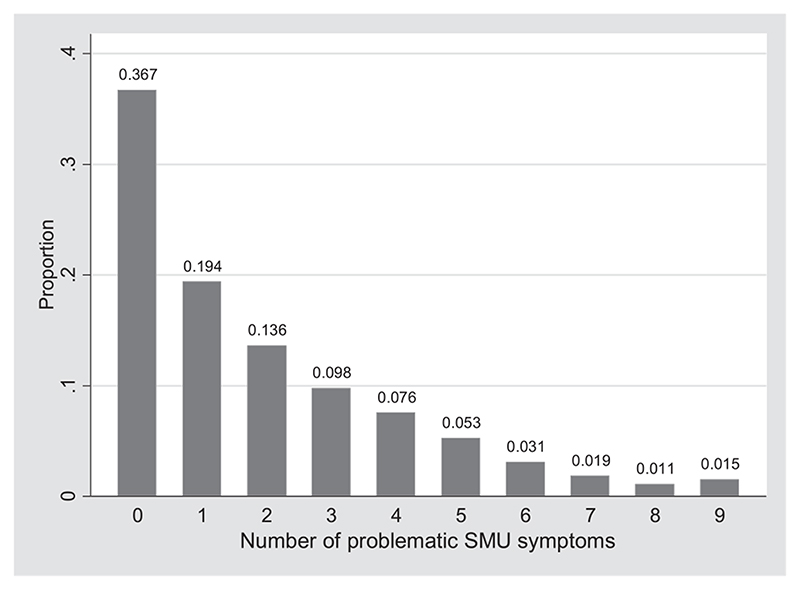
Distribution of the sum-score of the social media disorder scale, pooled sample n = 222 532. SMU = social media use

Figure 1 shows that more than a third of adolescents did not report symptoms, whereas 7.64% reported problematic SMU; that is, six or more symptoms. By country, problematic SMU ranged between 3.20% (the Netherlands) and 16.41% (Malta). All prevalence rates of problematic SMU (symptoms) by country can be found in the Supporting information, Table S2.

Multivariate logistic regressions were conducted to investigate whether problematic SMU differed by survey mode, gender, age and socio-economic status within each country. In none of the countries, problematic SMU differed by survey mode (Table 2). In multiple countries, gender, age and socio-economic status were associated with problematic SMU, although the direction of these associations was not consistent.

### Structural validity

As a preliminary step, EFAs were conducted prior to the CFAs. Details regarding the EFAs can be found in the Supporting information, Tables S3 and S4. Overall, 34 of 44 countries consistently showed that a one-factor model was preferred over a two- and three-factor model. In the 10 other countries, findings were inconsistent. However, the model fit of the one-factor model was good in all countries, as well as the quality of the factor. Thus, we consider the factor structure as unidimensional. As such, testing a one-factor model using CFA was considered justified.

CFAs showed that, in all countries, the one-factor model had good model fit (min. CFI and TLI = 0.963 and 0.951, max. RMSEA and SRMR = 0.057 and 0.060). On average (i.e., in the pooled sample), all factor loadings exceeded 0.50 (Table 3). In all countries, at least five factor loadings exceeded 0.50. More specifically, for 33 countries, all nine factor loadings exceeded 0.50. In nine countries, there was one item with a factor loading below 0.50. In two countries, there were two items with factor loadings below 0.50. However, the lowest observed factor loading was 0.38 (‘persistence’ in Greece). Details regarding the CFA estimated by country can be found in the Supporting information, Tables S5 and S6. Overall, the model fit and factor loadings confirm a solid structural validity in all countries.

### Reliability

Ordinal alpha for the nine items on the pooled sample was 0.90. Alpha ranged between 0.84 (Greece) and 0.95 (Azerbaijan), suggesting good reliability across all countries. Reliability estimates for all countries are provided in the Supporting information, Table S5.

### Measurement invariance

Table 4 shows that constraining the factor loadings and thresholds to be equal across countries did not substantially deteriorate model fit (ΔCFI = -0.010, ΔRMSEA = 0.003), indicating that the factor structure was comparable across countries. Given that the observed change in CFI was 0.10, which is the maximum value allowed for establishing measurement invariance [49], a sensitivity analysis was conducted. Specifically, the pooled sample was randomly split in half, after which the measurement invariance analysis was repeated using the two subsamples. For both subsamples, measurement invariance was established (for both subsamples: configural CFI = 0.981, RMSEA = 0.038; scalar CFI = 0.971, RMSEA = 0.041).

The pooled sample showed measurement invariance with respect to gender, age and socio-economic status (Table 4). By country, gender invariance was established in all countries, whereby the strongest decrease in CFI was observed in Greece and Hungary (ΔCFI = -0.006) and the strongest increase in RMSEA was observed in Greece (ΔRMSEA = 0.003). Age invariance was not established in Malta (ΔCFI = -0.013, ΔRMSEA = 0.008). In the other 43 countries, age invariance was established, whereby the highest decrease in CFI and increase in RMSEA was observed in Romania (ΔCFI = -0.007, ΔRMSEA = 0.004). Socio-economic invariance was established in all countries because CFI decreased with not more than 0.002 (Sweden) and RMSEA decreased in all countries with at least 0.001 (Kazakhstan). The invariance analyses by country are presented in the Supporting information, Tables S7-S9.

### Criterion validity

Table 5 shows the means in life satisfaction, psychosomatic complaints and intensity of online communication via social media, by problematic SMU, as well as the effect sizes of the mean differences. Although the outcome measures show skew distributions, it is unlikely that this significantly affects the results, because large samples were used [65]. Furthermore, mean differences were estimated using regression with MLR-estimation, which provides estimates robust to non-normality [66].

In the pooled sample, problematic users reported lower levels of life satisfaction, higher levels of psychosomatic complaints and higher online communication intensity than non-problematic users. The difference in life satisfaction and intensity of online communication between problematic and non-problematic users was small to moderate, whereas the difference in psychosomatic complaints was moderate to large (Table 5). The analyses by country showed that there was a negative association between problematic SMU and life satisfaction in 40 countries, with effect sizes ranging from small (Albania: *β* = −0.193, P = 0.021) to moderate/large (England: *β* = −0.682, P < 0.001). In four countries there were no significant differences in life satisfaction (Azerbaijan, Georgia, Kazakhstan and Republic of Moldova). The positive association between problematic SMU and psychosomatic complaints was observed in all countries, with effect sizes ranging from small/moderate (Norway: *β* = 0.309, P < 0.001) to large (Azerbaijan: *β* = 0.924, P < 0.001). The positive association between problematic SMU and the intensity of online communication was observed in 41 countries and ranged from small (Armenia: *β* = 0.163, P = 0.023) to moderate/large (Switzerland: *β* = 0.635, P < 0.001). In two countries (Georgia and the Russian Federation), there were no significant differences in the intensity of online communication. In one country, there was a small/moderate negative association between problematic SMU and the intensity of online communication (Azerbaijan: *β* = -0.273, P = 0.001). Estimates by country are presented in the Supporting information, Tables S10–S12.

Overall, for almost all countries, the associations were significant and in the expected directions, which confirms appropriate criterion validity. To investigate the robustness of this conclusion, we repeated the analyses while defining problematic SMU as reporting at least five or seven symptoms, instead of six. Results were highly comparable, suggesting that our findings were not sensitive to our operationalization of problematic SMU. A summary of this analysis is provided in the Supporting information, Table S13.

## Discussion

The present study is the first, to our knowledge, to systematically analyse the psychometric properties of a problematic SMU scale across comparable nationally representative samples of adolescents in many countries. Findings from 222 253 adolescents from 44 countries showed that the SMD scale has good psychometric properties within a broad international context, and demonstrates its suitability for cross-national comparisons in problematic SMU. First, the CFA confirmed good structural validity of the scale across all countries. Secondly, the internal consistency of the items was good in all countries, suggesting that the scale provides reliable scores. Thirdly, the factor structure of the scale was measurement invariant across countries. Also, gender and socio-economic status invariance was established in all countries and age invariance in all countries except Malta. Fourthly, in line with previous research, in almost all countries, problematic SMU was negatively associated with mental wellbeing and positively with the intensity of online communication, confirming good criterion validity.

All countries showed good structural validity by means of good model fit of a one-factor model and high factor loadings of the items. These findings suggest that all nine items substantially contribute to the underlying construct of problematic SMU. This implies that alongside the six items referring to the core criteria of addiction [4,12], the three additional items that distinguish the SMD scale from other problematic SMU scales [11,21], including problems, displacement and deception, further contribute to the conceptualization of problematic SMU. Hence, with their inclusion, the SMD scale may advance the measurement of problematic SMU. To verify this suggestion, future studies comparing the psychometric properties of the SMD scale with scales based on only the six core criteria of addiction are recommended.

The finding that the factor model was measurement invariant across countries implies that adolescents from different countries interpret the questions from the scale in a similar manner and that the scale measures the same underlying construct across countries [29]. Hence, the scale is suited for measuring and comparing adolescents’ level of problematic SMU in international surveys. Furthermore, as a next step, future research examining the potential reasons for country-level differences in the prevalence of problematic SMU are considered promising. Moreover, the finding that gender, age and socio-economic invariance was observed in all countries (except for age invariance in one country) implies that the scale also measures the same underlying construct for boys, girls, 11-, 13-, 15-year-olds and adolescents with low, middle and high socio-economic status. Therefore, researchers can use the scale to accurately identify which of these subgroups are at risk of problematic SMU, which is considered important given the possible detrimental consequences of problematic SMU [9,10].

The observed pooled effect sizes from the criterion validity analysis were in line with the literature [7,52–54]. Problematic SMU was more strongly associated with psychosomatic complaints than with low life satisfaction, which parallels review studies showing a stronger relationship between problematic SMU and indicators of negative mental wellbeing (e.g. depression) compared with indicators of positive mental wellbeing (e.g. self-esteem) [7,53]. Not only do these findings confirm that the scores on the scale are related to constructs to which they should theoretically be related; they also highlight that, world-wide, problematic users face several similar mental health risks. If these associations occur because problematic SMU leads to significant psychological harm, as suggested by some longitudinal studies [10,50], then problematic SMU may reflect addiction-like behaviour, which has been questioned [13]. To verify this, however, more research is required, particularly focusing upon whether problematic SMU impairs mental health and other aspects of daily life, assessed in clinical settings. Furthermore, the finding that problematic SMU is a global risk factor for adolescents’ mental wellbeing emphasizes the relevance for the development of prevention and intervention programmes on (reducing) problematic SMU; for example, by supporting adolescents in regulating their SMU.

In addition, the observed small-to-moderate effect size of the (positive) association between problematic SMU and online communication intensity may be regarded as counterintuitive [52]. However, this effect size is in line with earlier meta-analytical findings on the relationship between problematic SMU and the intensity of (tracked) SMU activities [52,54], which supports the suggestion that the intensity of SMU activities and problematic SMU should be regarded as related but different dimensions of SMU [10,32,54]. Although many problematic users may engage in a high intensity of online communication, there may also be problematic users who do not show intensive online communication. These latter users may experience a mismatch between their desired and actual online social network: they could be preoccupied with social media without having the desired network to interact with. Conversely, adolescents engaging in intensive online communication may be well able to regulate their online activities without experiencing problematic SMU.

### Strengths and limitations

The present study has several strengths related to the data that include many nationally representative subsamples. However, there are also some limitations that should be acknowledged. First, the cross-sectional design of the study precludes the possibility to investigate the predictive validity and the test-re-test reliability of the scale. Secondly, the present study included mainly European adolescents. Thirdly, other elements of validity, including convergent and discriminant validity, were not assessed. Considering these three limitations, more validation research on the SMD scale using longitudinal data and data from non-European adolescents, and including more validation analyses, is warranted to extend current knowledge on the psychometric properties of the scale. Fourthly, scores on the SMD scale are based on self-reports, which may deviate from assessments by others. As such, the reported prevalence rates of problematic SMU may be under- or overestimated. Research comparing self-report scores with scores from, for example, teachers or parents, is considered important. Fifthly, the evaluation criteria for measurement invariance testing were obtained from WLSMV estimation, which may not perform as well as with MLR estimation [67]. However, with categorical items, measurement invariance analysis with MLR estimation can only be conducted using χ^2^ difference tests, which may falsely reject measurement invariance due to its sensitivity to large sample sizes [48,49]. Sixthly, the present study defined adolescents reporting six or more symptoms as problematic users. Although this definition was based on findings from latent class analyses [15], research using clinical data is required to verify whether this definition adequately identifies problematic users; for example, by comparing assessments of problematic SMU by a clinician with assessments using our used definition based on the SMD scale. Finally, although response rates were generally high, results are possibly somewhat affected by voluntary response bias, given the sampling design.

## Conclusion

Given the widespread adoption of social media among adolescents and the risks that are associated with addiction-like problematic SMU observed world-wide, it is essential that a suitable measure is available to allow for adequate assessments and cross-national comparisons of problematic SMU. Findings from the present study demonstrate that the SMD scale is reliable, valid and comparable across many national contexts, thereby facilitating future research on problematic SMU.

## Supplementary Material

Supplementary Material

## Figures and Tables

**Table 1:** Prevalence problematic SMU symptoms (*n* = 222 532 in 44 countries)

During the past year, have you:	Item	%	Min. %^a^	Max. %^b^
Regularly found that you cannot think of anything else but the moment that you will be able to use social media again?	Preoccupation	22.07%	14.16%	34.73%
Regularly felt dissatisfied because you wanted to spend more time on social media?	Tolerance	18.89%	7.33%	35.34%
Often felt bad when you could not use social media?	Withdrawal	21.30%	11.63%	48.21%
Tried to spend less time on social media, but failed?	Persistence	30.66%	22.46%	42.10%
Regularly neglected other activities (e.g. hobbies, sport) because you wanted to use social media?	Displacement	15.73%	7.03%	26.13%
Regularly had arguments with others because of your social media use?	Problem	18.86%	11.87%	39.64%
Regularly lied to your parents or friends about the amount of time you spend on social media?	Deception	14.56%	8.76%	26.75%
Often used social media to escape from negative feelings?	Escape	30.74%	11.42%	47.02%
Had serious conflict with your parents, brother(s) or sister(s) because of your social media use?	Conflict	14.38%	4.67%	32.23%
Problematic SMU (six or more symptoms)		7.64%	3.20%	16.41%

SMU = social media use.

a Lowest observed prevalence across all 44 countries;

b highest observed prevalence across all 44 countries.

**Table 2:** Multivariate logistic regression, problematic SMU (*n* = 222 532 in 44 countries)

	Pooled sample			Analyses by country					
	B	SE	OR	Countries positive	Min. OR^a^	Max. OR^a^	Countries negative	Min. OR^b^	Max. OR^b^
Survey mode (ref. = paper-and-pencil self-completion)^c^									
Digital self-completion	−0.026	0.024	0.974	0			0		
Gender (ref. = boy)									
Girl	0.189***	0.019	1.208	19	1.326	1.853	4	0.475	0.779
Age (ref. = 11-year-old)									
13-year-old	0.394***	0.028	1.484	27	1.395	3.225	1	0.215	0.215
15-year-old	0.477***	0.029	1.612	28	1.470	3.238	1	0.341	0.341
Socio-economic status (ref. = low)									
Middle	−0.100***	0.023	0.905	1	2.939	2.939	4	0.576	0.683
High	−0.023	0.028	0.977	1	1.547	1.547	5	0.503	0.682

SMU = social media use; B = logit coefficient; SE = standard error; OR = odds ratio; ref. = reference category;

*** P < 0.001; problematic SMU was defined as reporting six to nine problematic SMU criteria.

a Minimum/maximum value of the OR across countries where a positive association was found (P < 0.05);

b minimum/maximum value of the OR across countries where a negative association was found (P < 0.05);

c the association between survey mode and problematic SMU was estimated across eight of 44 countries (n = 43 802), because there were only eight countries where both survey modes were employed.

**Table 3:** Summary CFA results, validation samples by country (*n* = 111 278 in 44 countries)

During the past year, have you:	Item	Min. loading^a^	Max. loading^b^	Average loading^c^
Regularly found that you cannot think of anything else but the moment that you will be able to use social media again?	Preoccupation	0.524	0.805	0.709
Regularly felt dissatisfied because you wanted to spend more time on social media?	Tolerance	0.630	0.857	0.743
Often felt bad when you could not use social media?	Withdrawal	0.604	0.851	0.733
Tried to spend less time on social media, but failed?	Persistence	0.380	0.814	0.566
Regularly neglected other activities (e.g. hobbies, sport) because you wanted to use social media?	Displacement	0.509	0.838	0.654
Regularly had arguments with others because of your social media use?	Problem	0.470	0.873	0.718
Regularly lied to your parents or friends about the amount of time you spend on social media?	Deception	0.589	0.859	0.738
Often used social media to escape from negative feelings?	Escape	0.496	0.829	0.615
Had serious conflict with your parents, brother(s) or sister(s) because of your social media use?	Conflict	0.617	0.930	0.766

CFA = confirmatory factor analysis.

a Lowest observed factor loading across all 44 countries;

b highest observed factor loading across all 44 countries;

c average factor loading calculated from 44 countries.

**Table 4:** Summary table measurement invariance analysis (*n* = 222 532 in 44 countries)

	Model fit					Change in model fit	
	Par.	CFI	TLI	RMSEA	SRMR	ΔCFI	ΔRMSEA
Country invariance							
Configural	792	0.979	0.972	0.037	0.040		
Scalar	491	0.969	0.967	0.040	0.045	−0.010	0.003
Gender invariance							
Configural	36	0.979	0.972	0.035	0.034		
Scalar	29	0.978	0.974	0.034	0.034	−0.001	−0.001
By country, minimum						−0.006	
By country, maximum							0.003
Age invariance^a^							
Configural	54	0.975	0.967	0.035	0.034		
Scalar	40	0.974	0.970	0.033	0.034	−0.001	−0.002
By country, minimum						−0.013	
By country, maximum							0.008
Socio-economic invariance^b^							
Configural	54	0.981	0.975	0.035	0.033		
Scalar	40	0.982	0.979	0.032	0.033	0.001	−0.003
By country, minimum						−0.002	
By country, maximum							−0.001

Par. = number of free parameters; CFI = comparative fit index; TLI = Tucker Lewis index; RMSEA = root mean square error of approximation; SRMR = standardized root mean square residual.

a *n* = 221 093 due to missing values of age;

b *n* = 212 353 due to missing values of socio-economic status.

**Table 5:** Summary table life satisfaction, psychosomatic complaints, and intensity of online communication by problematic SMU (*n* = 222 532 in 44 countries)

	Means					Effect size mean differences					
	Mean	95% LL	95% UL	Observed range mean^a^		*β*	SE	P	Countries^b^	Observed range *β*^c^	
	Life satisfaction (mean = 7.73, SD = 2.03, min. = 0, max. = 10)										
Non-problematic	7.79	7.79	7.80	6.67	8.56						
Problematic	6.96	6.92	7.00	6.13	8.30	−0.395	0.011	< 0.001	40	−0.682	−0.193
	Psychosomatic complaints (mean = 2.08, SD = 0.90, min. = 1, max. = 5)										
Non-problematic	2.03	2.03	2.04	1.60	2.39						
Problematic	2.62	2.60	2.63	2.06	3.26	0.648	0.010	< 0.001	44	0.309	0.924
	Intensity of online communication (mean = 3.76, SD = 1.29, min. = 1, max. = 5)										
Non-problematic	3.72	3.72	3.73	2.84	4.12						
Problematic	4.15	4.13	4.17	2.33	4.45	0.313	0.009	< 0.001	41	0.163	0.635

SMU = social media use; LL = confidence interval lower limit; UL = confidence interval upper limit; *β* = STDY-standardized [i.e. B/standard deviation (Y)], controlled for gender, age and socio-economic status; SE = standard error.

a Observed means across 44 countries;

b number of countries where a significant association was observed in the same direction as in the pooled sample;

c observed range STDY-standardized β across countries where a significant association was observed in the same direction as in the pooled sample, controlled for gender, age and socio-economic status.
