# Supplementary material for: Cross-national validation of the social media disorder scale: findings from adolescents from 44 countries
Source: Addiction. Author manuscript; Available in PMC 2023 Jan 9. (PMC7614030; doi:10.1111/add.15709)
Supplement: Supplementary Material [file EMS157717-supplement-Supplementary_Material.docx]

**Cross-national validation of the Social Media Disorder-scale: Findings from adolescents from 44 countries**

Maartje Boer, Regina J. J. M. van den Eijnden, Catrin Finkenauer, Meyran Boniel-Nissim, Claudia Marino, Jo Inchley, Alina Cosma, Leena Paakkari, Gonneke W.J.M. Stevens

**ONLINE SUPPLEMENT**

**Note**

Codes for data preparation, imputation of missing data, and analyses can be consulted via <https://osf.io/bgkec/>

| **Table S1: The Social Media Disorder Scale** | | |
| --- | --- | --- |
| **We are interested in your experiences with social media. The term social media refers to social network sites (e.g., Facebook, [add other local examples]) and instant messengers (e.g., [insert local examples], WhatsApp, Snapchat, Facebook messenger).**  **During the past year, have you… *Please tick one circle for each line.*** | | |
|  | **No** | **Yes** |
| …regularly found that you can’t think of anything else but the moment that you will be able to use social media again? | O | O |
| …regularly felt dissatisfied because you wanted to spend more time on social media? | O | O |
| …often felt bad when you could not use social media? | O | O |
| …tried to spend less time on social media, but failed? | O | O |
| …regularly neglected other activities (e.g., hobbies, sport) because you wanted to use social media? | O | O |
| …regularly had arguments with others because of your social media use? | O | O |
| …regularly lied to your parents or friends about the amount of time you spend on social media? | O | O |
| …often used social media to escape from negative feelings? | O | O |
| …had serious conflict with your parents, brother(s) or sister(s) because of your social media use? | O | O |
| **Scoring instructions for assessor:**  From top to bottom, the nine items represent the following criteria: preoccupation, tolerance, withdrawal, persistence, displacement, problem, deception, escape, and conflict[1]. Respondents with six to nine *yes*-responses may be coded as ‘problematic user’, and respondents with zero to five *yes*-responses as ‘non-problematic user’[2]. | | |

| **Table S2: Prevalence rates items problematic SMU, by country (n = 222,532 in 44 countries)** | | | | | | | | | | | |
| --- | --- | --- | --- | --- | --- | --- | --- | --- | --- | --- | --- |
|  |  | **Problematic SMU items** | | | | | | | | | **Probl. SMU^1^** |
|  | ***n*** | **(1)** | **(2)** | **(3)** | **(4)** | **(5)** | **(6)** | **(7)** | **(8)** | **(9)** |  |
| Albania | 1650 | 23.43% | 22.19% | 35.53% | **41.13%** | 23.84% | 39.64% | *16.96%* | 31.82% | 19.85% | 11.71% |
| Armenia | 4430 | 16.28% | 14.19% | 21.45% | **23.58%** | 14.38% | 16.39% | *11.49%* | 20.80% | 13.88% | 6.08% |
| Austria | 4011 | 20.14% | 15.93% | 18.32% | 28.54% | 17.68% | *11.87%* | 12.98% | **29.18%** | 14.94% | 4.85% |
| Azerbaijan | 3778 | 25.73% | 21.56% | 20.12% | **24.89%** | 18.31% | 18.06% | *15.68%* | 22.72% | 17.09% | 9.99% |
| Belgium (Flanders) | 4117 | 28.04% | 16.79% | 18.63% | **30.78%** | 15.36% | 18.97% | 16.12% | 29.05% | *13.40%* | 6.73% |
| Belgium (Wallonia) | 5221 | 23.60% | 24.98% | 24.46% | 29.95% | *14.84%* | 23.36% | 18.13% | **32.43%** | 18.03% | 8.62% |
| Canada | 12355 | 18.30% | 15.46% | 17.18% | 27.70% | 12.51% | 15.41% | 13.77% | **31.34%** | *10.29%* | 6.28% |
| Croatia | 4913 | 26.22% | 19.94% | 24.49% | **38.54%** | 19.18% | 20.99% | 16.57% | 30.86% | *14.99%* | 10.31% |
| Czechia | 11162 | 16.40% | 14.40% | 18.44% | 26.51% | 13.57% | 15.21% | *10.82%* | **27.29%** | 12.48% | 5.26% |
| Denmark | 3113 | 25.36% | 8.13% | 13.83% | 26.82% | *7.03%* | 13.48% | 9.39% | **31.41%** | 7.92% | 3.91% |
| England | 3306 | 19.88% | 18.31% | 23.57% | 31.35% | 13.75% | 18.58% | 13.77% | **34.22%** | *11.56%* | 7.77% |
| Estonia | 4622 | 19.13% | 19.07% | 21.39% | 29.45% | 11.08% | 14.50% | *10.83%* | **35.18%** | 12.26% | 5.81% |
| Finland | 3067 | 34.73% | 18.34% | 19.38% | **31.41%** | 18.78% | 17.46% | *12.94%* | 30.87% | 14.44% | 10.02% |
| France | 8621 | 22.66% | 22.86% | 22.00% | **26.35%** | *11.42%* | 19.37% | 17.10% | 26.32% | 13.22% | 7.41% |
| Georgia | 4067 | 18.65% | 19.91% | 21.76% | **31.83%** | 18.76% | 13.89% | *9.46%* | 11.42% | 10.10% | 4.33% |
| Germany | 4126 | 17.61% | 17.51% | 19.60% | **29.92%** | 14.98% | 14.05% | *13.06%* | 26.05% | 18.74% | 5.28% |
| Greece | 3715 | 23.67% | 23.28% | 30.78% | 32.09% | *15.14%* | 24.15% | 18.69% | **42.13%** | 22.01% | 9.98% |
| Hungary | 3715 | 16.66% | 21.22% | 19.81% | 27.80% | 15.46% | 24.55% | *9.27%* | **30.76%** | 13.54% | 5.38% |
| Iceland | 6693 | 14.97% | 12.59% | 11.63% | **28.70%** | *7.11%* | 13.64% | 12.25% | 18.60% | 7.94% | 4.66% |
| Ireland | 3628 | 28.42% | 23.21% | 31.04% | 37.43% | *14.77%* | 28.20% | 23.26% | **40.62%** | 14.80% | 12.01% |
| Israel | 7134 | 24.12% | 17.19% | 14.40% | **23.08%** | 17.03% | 15.14% | 8.76% | 19.24% | *8.02%* | 5.02% |
| Italy | 4069 | 28.80% | *16.93%* | 22.68% | 37.09% | 22.56% | 28.71% | 25.88% | **38.84%** | 24.52% | 10.86% |
| Kazakhstan | 4488 | 17.09% | 13.07% | 13.75% | **22.46%** | 15.95% | 12.05% | *9.54%* | 19.65% | 9.76% | 4.38% |
| Latvia | 4143 | 17.08% | 15.04% | 22.22% | **36.08%** | 14.13% | 13.65% | *11.49%* | 34.89% | 12.95% | 5.31% |
| Lithuania | 3685 | 21.15% | 18.28% | 19.52% | 30.14% | 15.47% | 15.92% | *12.53%* | **31.65%** | 16.98% | 7.78% |
| Luxembourg | 3889 | 22.57% | 20.82% | 25.55% | **33.48%** | *16.60%* | 17.35% | 16.62% | 31.96% | 19.90% | 7.56% |
| Malta | 2504 | 32.04% | 35.34% | **48.21%** | 42.10% | *21.18%* | 33.40% | 22.31% | 35.39% | 32.23% | 16.41% |
| Netherlands | 4579 | 26.10% | 7.33% | 15.70% | 24.52% | 13.14% | 14.26% | 10.99% | **28.41%** | *4.67%* | 3.20% |
| North Macedonia | 4575 | 31.16% | 25.63% | 32.48% | 36.41% | 26.13% | 17.21% | *12.17%* | **47.02%** | 14.97% | 9.55% |
| Norway | 3053 | 21.57% | 17.65% | 21.97% | 29.34% | 16.06% | 19.24% | 17.27% | **30.27%** | *12.91%* | 9.19% |
| Poland | 5055 | 22.83% | 20.68% | 26.31% | 24.87% | 17.18% | 20.83% | 16.32% | **32.85%** | *14.77%* | 7.62% |
| Portugal | 5866 | 19.40% | 17.59% | 20.58% | 25.44% | *9.42%* | 14.99% | 9.53% | **27.25%** | 10.96% | 5.86% |
| Republic of Moldova | 4429 | 23.86% | 24.34% | 24.14% | 34.18% | 22.28% | 19.87% | *15.72%* | **40.95%** | 18.37% | 7.95% |
| Romania | 4483 | 27.99% | 28.29% | 27.74% | 37.66% | 25.76% | 28.65% | 25.58% | **45.41%** | *18.90%* | 13.04% |
| Russian Federation | 4061 | 19.98% | 16.49% | 16.23% | 25.56% | 18.53% | 15.44% | *11.76%* | **28.62%** | 18.03% | 7.65% |
| Scotland | 4916 | 23.42% | 19.46% | 25.65% | **35.03%** | 15.25% | 23.08% | 15.16% | 34.18% | *11.61%* | 9.36% |
| Serbia | 3740 | 18.10% | 17.09% | 15.82% | **31.52%** | 16.52% | 15.84% | 13.79% | 31.43% | *12.65%* | 6.96% |
| Slovenia | 5126 | 14.16% | *11.74%* | 15.47% | **30.96%** | 12.93% | 13.50% | 13.00% | 21.96% | 13.24% | 5.24% |
| Spain | 4070 | 27.86% | 29.63% | 28.39% | **35.66%** | 24.38% | 33.77% | 26.75% | 33.45% | *22.68%* | 14.27% |
| Sweden | 4006 | 20.15% | 15.43% | 14.99% | 31.53% | *8.55%* | 14.61% | 12.60% | **35.44%** | 10.18% | 5.17% |
| Switzerland | 7122 | 17.10% | 16.53% | 15.63% | **30.36%** | *12.40%* | 16.66% | 12.86% | 27.53% | 16.33% | 4.48% |
| Turkey | 5541 | 25.14% | 30.23% | 25.17% | 33.35% | *17.01%* | 23.92% | 19.02% | **35.39%** | 24.11% | 10.70% |
| Ukraine | 6232 | 23.69% | 18.79% | 15.01% | **36.67%** | 17.38% | 16.69% | *10.53%* | 36.13% | 13.38% | 6.79% |
| Wales | 15456 | 25.88% | 21.16% | 26.45% | 35.82% | 18.33% | 24.94% | 17.58% | **35.93%** | *15.12%* | 12.27% |
| Notes: SMU = social media use; (1) = preoccupation; (2) = tolerance; (3) = withdrawal; (4) = persistence; (5) = displacement; (6) = problem; (7) = deception; (8) = escape; (9) = conflict. Rates in italics indicate the row minimum with respect to the nine items; rates in boldface indicate the row maximum with respect to the nine items.  ^1^ Problematic SMU, i.e., presence of six to nine symptoms. The present prevalence estimates for problematic SMU slightly differ from previously reported prevalence estimates using the same data[3] (*M*Δ = -0.01 percentage point, min. = -0.29 in Italy, max. = +0.42 in Canada). These small differences are the result of improved estimation of missing data due to inclusion of more data (countries) in the present study. Also, the present prevalence estimate of the Belgian (Wallonia) sample deviates more from the previous report[3] (+0.6 percentage point), because the present study uses a larger subsample that became available in a later release of the data. | | | | | | | | | | | |

| **Table S3: EFA eigenvalues and parallel test results, calibration samples, by country (n = 111,254 in 44 countries)** | | | | | | | | | | | | | | | | | | | |
| --- | --- | --- | --- | --- | --- | --- | --- | --- | --- | --- | --- | --- | --- | --- | --- | --- | --- | --- | --- |
| **Country** | ***n*** | **Empirical eigenvalues^1^** | | | | | | | | | **95^th^ percentile of random eigenvalues^2^** | | | | | | | | |
|  |  | **1** | **2** | **3** | **4** | **5** | **6** | **7** | **8** | **9** | **1** | **2** | **3** | **4** | **5** | **6** | **7** | **8** | **9** |
| Albania | 825 | **4.41** | 0.86 | 0.80 | 0.67 | 0.64 | 0.49 | 0.44 | 0.37 | 0.33 | *1.21* | 1.14 | 1.10 | 1.06 | 1.02 | 0.99 | 0.96 | 0.92 | 0.88 |
| Armenia | 2215 | **5.07** | 0.76 | 0.67 | 0.64 | 0.46 | 0.43 | 0.39 | 0.33 | 0.25 | *1.13* | 1.09 | 1.06 | 1.04 | 1.01 | 0.99 | 0.97 | 0.95 | 0.93 |
| Austria | 2005 | **4.14** | 0.86 | 0.77 | 0.69 | 0.62 | 0.56 | 0.52 | 0.44 | 0.40 | *1.13* | 1.09 | 1.06 | 1.04 | 1.01 | 0.99 | 0.97 | 0.95 | 0.93 |
| Azerbaijan | 1889 | **6.50** | 0.67 | 0.39 | 0.36 | 0.34 | 0.24 | 0.20 | 0.17 | 0.13 | *1.14* | 1.09 | 1.06 | 1.04 | 1.02 | 0.99 | 0.97 | 0.95 | 0.92 |
| Belgium (Flanders) | 2058 | **4.62** | 0.85 | 0.75 | 0.60 | 0.55 | 0.49 | 0.43 | 0.37 | 0.34 | *1.13* | 1.09 | 1.06 | 1.04 | 1.01 | 0.99 | 0.97 | 0.95 | 0.93 |
| Belgium (Wallonia) | 2610 | **4.43** | 0.87 | 0.73 | 0.67 | 0.65 | 0.48 | 0.43 | 0.40 | 0.33 | *1.12* | 1.08 | 1.06 | 1.03 | 1.01 | 1.00 | 0.98 | 0.96 | 0.93 |
| Canada | 6177 | **4.99** | 0.87 | 0.74 | 0.56 | 0.52 | 0.43 | 0.36 | 0.28 | 0.26 | *1.07* | 1.05 | 1.04 | 1.02 | 1.01 | 1.00 | 0.98 | 0.97 | 0.96 |
| Croatia | 2456 | **5.10** | 0.83 | 0.69 | 0.53 | 0.52 | 0.39 | 0.36 | 0.32 | 0.28 | *1.12* | 1.08 | 1.06 | 1.03 | 1.01 | 0.99 | 0.98 | 0.95 | 0.93 |
| Czechia | 5581 | **4.94** | 0.78 | 0.73 | 0.61 | 0.54 | 0.41 | 0.38 | 0.33 | 0.29 | *1.08* | 1.06 | 1.04 | 1.02 | 1.01 | 1.00 | 0.98 | 0.97 | 0.96 |
| Denmark | 1556 | **4.89** | 0.88 | 0.79 | 0.66 | 0.57 | 0.42 | 0.33 | 0.25 | 0.22 | *1.15* | 1.10 | 1.07 | 1.04 | 1.02 | 0.99 | 0.97 | 0.94 | 0.91 |
| England | 1653 | **5.31** | 0.75 | 0.66 | 0.55 | 0.53 | 0.36 | 0.34 | 0.27 | 0.23 | *1.15* | 1.10 | 1.07 | 1.04 | 1.02 | 0.99 | 0.97 | 0.95 | 0.92 |
| Estonia | 2311 | **4.66** | 0.86 | 0.76 | 0.65 | 0.60 | 0.47 | 0.42 | 0.33 | 0.25 | *1.13* | 1.08 | 1.06 | 1.03 | 1.01 | 0.99 | 0.97 | 0.95 | 0.93 |
| Finland | 1533 | **5.91** | 0.69 | 0.53 | 0.47 | 0.38 | 0.37 | 0.26 | 0.21 | 0.19 | *1.16* | 1.11 | 1.07 | 1.04 | 1.02 | 0.99 | 0.97 | 0.94 | 0.91 |
| France | 4310 | **4.80** | 0.79 | 0.69 | 0.62 | 0.56 | 0.53 | 0.39 | 0.35 | 0.28 | *1.09* | 1.06 | 1.04 | 1.03 | 1.01 | 1.00 | 0.98 | 0.97 | 0.95 |
| Georgia | 2033 | **4.76** | 0.86 | 0.71 | 0.68 | 0.51 | 0.43 | 0.42 | 0.38 | 0.25 | *1.13* | 1.09 | 1.06 | 1.04 | 1.01 | 0.99 | 0.97 | 0.95 | 0.92 |
| Germany | 2063 | **4.51** | 0.85 | 0.73 | 0.66 | 0.64 | 0.47 | 0.44 | 0.37 | 0.34 | *1.13* | 1.09 | 1.06 | 1.04 | 1.01 | 0.99 | 0.97 | 0.95 | 0.92 |
| Greece | 1857 | **4.19** | 0.85 | 0.81 | 0.67 | 0.62 | 0.57 | 0.48 | 0.44 | 0.37 | *1.14* | 1.09 | 1.06 | 1.04 | 1.01 | 0.99 | 0.97 | 0.95 | 0.92 |
| Hungary | 1857 | **4.11** | 0.92 | 0.89 | 0.71 | 0.68 | 0.51 | 0.45 | 0.38 | 0.36 | *1.14* | 1.09 | 1.06 | 1.04 | 1.01 | 0.99 | 0.97 | 0.95 | 0.92 |
| Iceland | 3346 | **5.86** | 0.69 | 0.58 | 0.44 | 0.39 | 0.35 | 0.28 | 0.22 | 0.19 | *1.10* | 1.07 | 1.05 | 1.03 | 1.01 | 0.99 | 0.98 | 0.96 | 0.94 |
| Ireland | 1814 | **4.75** | 0.95 | 0.72 | 0.64 | 0.56 | 0.44 | 0.38 | 0.29 | 0.27 | *1.14* | 1.10 | 1.07 | 1.04 | 1.02 | 0.99 | 0.97 | 0.95 | 0.92 |
| Israel | 3567 | **4.61** | 0.98 | 0.65 | 0.62 | 0.56 | 0.48 | 0.43 | 0.39 | 0.29 | *1.10* | 1.07 | 1.05 | 1.03 | 1.01 | 1.00 | 0.98 | 0.96 | 0.94 |
| Italy | 2034 | **4.13** | 0.85 | 0.83 | 0.67 | 0.67 | 0.52 | 0.49 | 0.47 | 0.38 | *1.13* | 1.09 | 1.06 | 1.04 | 1.01 | 0.99 | 0.97 | 0.95 | 0.93 |
| Kazakhstan | 2244 | **5.17** | 0.75 | 0.66 | 0.54 | 0.46 | 0.45 | 0.37 | 0.34 | 0.27 | *1.13* | 1.09 | 1.06 | 1.03 | 1.01 | 0.99 | 0.97 | 0.95 | 0.93 |
| Latvia | 2071 | **4.47** | 0.90 | 0.77 | 0.70 | 0.60 | 0.49 | 0.42 | 0.38 | 0.28 | *1.13* | 1.09 | 1.06 | 1.04 | 1.02 | 0.99 | 0.97 | 0.95 | 0.93 |
| Lithuania | 1842 | **5.17** | 0.80 | 0.71 | 0.60 | 0.45 | 0.42 | 0.33 | 0.30 | 0.24 | *1.14* | 1.10 | 1.06 | 1.04 | 1.01 | 0.99 | 0.97 | 0.95 | 0.92 |
| Luxembourg | 1944 | **4.10** | 0.87 | 0.78 | 0.76 | 0.68 | 0.53 | 0.51 | 0.42 | 0.37 | *1.14* | 1.09 | 1.06 | 1.04 | 1.02 | 0.99 | 0.97 | 0.95 | 0.92 |
| Malta | 2287 | **4.32** | 0.87 | 0.84 | 0.70 | 0.66 | 0.53 | 0.42 | 0.34 | 0.32 | *1.13* | 1.09 | 1.06 | 1.03 | 1.01 | 0.99 | 0.97 | 0.95 | 0.93 |
| Netherlands | 1252 | **4.32** | 0.76 | 0.75 | 0.71 | 0.65 | 0.54 | 0.49 | 0.42 | 0.35 | *1.17* | 1.12 | 1.08 | 1.05 | 1.02 | 0.99 | 0.96 | 0.93 | 0.91 |
| North Macedonia | 2289 | **4.36** | 0.79 | 0.76 | 0.69 | 0.64 | 0.61 | 0.44 | 0.40 | 0.31 | *1.13* | 1.09 | 1.06 | 1.03 | 1.01 | 0.99 | 0.98 | 0.95 | 0.93 |
| Norway | 1526 | **5.73** | 0.72 | 0.59 | 0.54 | 0.39 | 0.33 | 0.31 | 0.25 | 0.14 | *1.15* | 1.11 | 1.07 | 1.04 | 1.02 | 0.99 | 0.97 | 0.94 | 0.91 |
| Poland | 2527 | **4.48** | 0.86 | 0.76 | 0.66 | 0.56 | 0.50 | 0.44 | 0.39 | 0.35 | *1.12* | 1.08 | 1.05 | 1.03 | 1.01 | 0.99 | 0.98 | 0.96 | 0.93 |
| Portugal | 2933 | **5.35** | 0.85 | 0.60 | 0.49 | 0.45 | 0.36 | 0.33 | 0.31 | 0.27 | *1.11* | 1.08 | 1.05 | 1.03 | 1.01 | 0.99 | 0.98 | 0.96 | 0.94 |
| Republic of Moldova | 2214 | **3.80** | 0.88 | 0.80 | 0.70 | 0.69 | 0.62 | 0.61 | 0.51 | 0.38 | *1.13* | 1.09 | 1.06 | 1.03 | 1.01 | 0.99 | 0.97 | 0.95 | 0.93 |
| ^1^ Derived from the tetrachoric correlation matrix as appropriate for categorical items.  ^2^ Results from 1000 randomly generated eigenvalues.  Notes: EFA = exploratory factor analysis; the EFA and parallel analysis were conducted using the Mplus default settings for EFA with categorical indicators (using Oblique Geomin rotation); Boldface numbers denote eigenvalues higher than one; Italics numbers denote 95 percentile values that exceeded the respective empirical eigenvalues. | | | | | | | | | | | | | | | | | | | |

| **Table S3 (continued): EFA eigenvalues and parallel test results, calibration samples, by country (n = 111,254 in 44 countries)** | | | | | | | | | | | | | | | | | | | |
| --- | --- | --- | --- | --- | --- | --- | --- | --- | --- | --- | --- | --- | --- | --- | --- | --- | --- | --- | --- |
| **Country** | ***n*** | **Empirical eigenvalues^1^** | | | | | | | | | **95^th^ percentile of random eigenvalues^2^** | | | | | | | | |
|  |  | **1** | **2** | **3** | **4** | **5** | **6** | **7** | **8** | **9** | **1** | **2** | **3** | **4** | **5** | **6** | **7** | **8** | **9** |
| Romania | 2241 | **4.06** | 0.88 | 0.83 | 0.70 | 0.66 | 0.59 | 0.49 | 0.44 | 0.37 | *1.13* | 1.09 | 1.06 | 1.03 | 1.01 | 0.99 | 0.97 | 0.95 | 0.93 |
| Russian Federation | 2030 | **5.57** | 0.66 | 0.60 | 0.48 | 0.43 | 0.39 | 0.37 | 0.26 | 0.23 | *1.13* | 1.09 | 1.06 | 1.04 | 1.01 | 0.99 | 0.97 | 0.95 | 0.92 |
| Scotland | 2458 | **5.04** | 0.88 | 0.66 | 0.58 | 0.52 | 0.43 | 0.39 | 0.30 | 0.20 | *1.12* | 1.08 | 1.06 | 1.03 | 1.01 | 0.99 | 0.98 | 0.96 | 0.93 |
| Serbia | 1870 | **5.09** | 0.88 | 0.76 | 0.62 | 0.47 | 0.38 | 0.34 | 0.30 | 0.17 | *1.14* | 1.10 | 1.06 | 1.04 | 1.01 | 0.99 | 0.97 | 0.95 | 0.92 |
| Slovenia | 2563 | **5.23** | 0.83 | 0.61 | 0.53 | 0.46 | 0.39 | 0.36 | 0.32 | 0.28 | *1.12* | 1.08 | 1.05 | 1.03 | 1.01 | 0.99 | 0.98 | 0.96 | 0.93 |
| Spain | 2035 | **5.32** | 0.75 | 0.60 | 0.57 | 0.48 | 0.39 | 0.36 | 0.31 | 0.21 | *1.13* | 1.09 | 1.06 | 1.04 | 1.01 | 0.99 | 0.97 | 0.95 | 0.93 |
| Sweden | 2003 | **4.61** | **1.03** | 0.69 | 0.66 | 0.57 | 0.43 | 0.40 | 0.36 | 0.26 | *1.13* | 1.09 | 1.06 | 1.04 | 1.01 | 0.99 | 0.97 | 0.95 | 0.92 |
| Switzerland | 3561 | **4.06** | 0.83 | 0.75 | 0.72 | 0.66 | 0.59 | 0.52 | 0.45 | 0.42 | *1.10* | 1.07 | 1.05 | 1.03 | 1.01 | 0.99 | 0.98 | 0.96 | 0.94 |
| Turkey | 2770 | **4.63** | 0.82 | 0.69 | 0.58 | 0.53 | 0.51 | 0.46 | 0.43 | 0.34 | *1.11* | 1.08 | 1.05 | 1.03 | 1.01 | 0.99 | 0.98 | 0.96 | 0.94 |
| Ukraine | 3116 | **4.70** | **1.01** | 0.68 | 0.65 | 0.50 | 0.46 | 0.38 | 0.36 | 0.27 | *1.11* | 1.07 | 1.05 | 1.03 | 1.01 | 1.00 | 0.98 | 0.96 | 0.94 |
| Wales | 7728 | **5.49** | 0.71 | 0.63 | 0.53 | 0.43 | 0.39 | 0.32 | 0.29 | 0.23 | *1.07* | 1.05 | 1.03 | 1.02 | 1.01 | 1.00 | 0.99 | 0.98 | 0.96 |
| ^1^ Derived from the tetrachoric correlation matrix as appropriate for categorical items.  ^2^ Results from 1000 randomly generated eigenvalues.  Notes: EFA = exploratory factor analysis; the EFA and parallel analysis were conducted using the Mplus default settings for EFA with categorical indicators (using Oblique Geomin rotation); Boldface numbers denote eigenvalues higher than one; Italics numbers denote 95 percentile values that exceeded the respective empirical eigenvalues. | | | | | | | | | | | | | | | | | | | |

| **Table S4: EFA factor solutions, calibration samples, by country (n = 111,254 in 44 countries)** | | | | | | | | | | | | | | | | | |
| --- | --- | --- | --- | --- | --- | --- | --- | --- | --- | --- | --- | --- | --- | --- | --- | --- | --- |
|  |  |  | **Model fit** | | | | | **Factor loadings** | | | | | | | | | |
| **Country** | **Model** | ***n*** | **Par.** | **CFI** | **TLI** | **RMSEA** | **SRMR** | **Factor** | **(1)** | **(2)** | **(3)** | **(4)** | **(5)** | **(6)** | **(7)** | **(8)** | **(9)** |
| Albania | m1 | 825 | 18 | 0.986 | 0.982 | 0.031 | 0.043 | 1 | **0.617** | **0.761** | **0.744** | **0.584** | **0.632** | **0.488** | **0.703** | **0.660** | **0.696** |
|  | m2 | 825 | 26 | - | - | - | - | - |  |  |  |  |  |  |  |  |  |
|  | m3 | 825 | 33 | - | - | - | - | - |  |  |  |  |  |  |  |  |  |
| Armenia | m1 | 2215 | 18 | 0.985 | 0.980 | 0.034 | 0.041 | 1 | **0.719** | **0.737** | **0.739** | **0.582** | **0.759** | **0.714** | **0.812** | **0.612** | **0.761** |
|  | m2 | 2215 | 26 | - | - | - | - | - |  |  |  |  |  |  |  |  |  |
|  | m3 | 2215 | 33 | - | - | - | - | - |  |  |  |  |  |  |  |  |  |
| Austria | m1 | 2005 | 18 | 0.987 | 0.982 | 0.025 | 0.036 | 1 | **0.681** | **0.646** | **0.702** | **0.567** | **0.533** | **0.644** | **0.661** | **0.517** | **0.680** |
|  | m2 | 2005 | 26 | - | - | - | - | - |  |  |  |  |  |  |  |  |  |
|  | m3 | 2005 | 33 | 1.000 | 1.002 | 0.000 | 0.015 | 1 | **0.563** | **0.746** | **0.897** | 0.003 | **0.273** | **0.321** | **0.348** | 0.306 | 0.000 |
|  |  |  |  |  |  |  |  | 2 | 0.064 | -0.011 | -0.223 | **0.611** | 0.033 | -0.004 | 0.012 | **0.360** | -0.026 |
|  |  |  |  |  |  |  |  | 3 | 0.111 | -0.070 | 0.003 | 0.251 | **0.286** | **0.388** | **0.365** | -0.003 | **0.855** |
| Azerbaijan | m1 | 1889 | 18 | 0.980 | 0.973 | 0.044 | 0.043 | 1 | **0.724** | **0.811** | **0.845** | **0.825** | **0.858** | **0.882** | **0.865** | **0.848** | **0.897** |
|  | m2 | 1889 | 26 | - | - | - | - | - |  |  |  |  |  |  |  |  |  |
|  | m3 | 1889 | 33 | - | - | - | - | - |  |  |  |  |  |  |  |  |  |
| Belgium (Flanders) | m1 | 2058 | 18 | 0.984 | 0.979 | 0.033 | 0.038 | 1 | **0.688** | **0.739** | **0.762** | **0.477** | **0.594** | **0.694** | **0.695** | **0.631** | **0.731** |
|  | m2 | 2058 | 26 | 0.998 | 0.997 | 0.012 | 0.019 | 1 | **0.706** | **0.863** | **0.757** | **0.398** | **0.589** | 0.089 | -0.006 | **0.242** | -0.003 |
|  |  |  |  |  |  |  |  | 2 | 0.011 | -0.094 | 0.036 | 0.100 | 0.027 | **0.658** | **0.757** | **0.425** | **0.790** |
|  | m3 | 2058 | 33 | 1.000 | 1.000 | 0.004 | 0.014 | 1 | **0.667** | **0.822** | **0.771** | 0.270 | **0.586** | 0.029 | 0.007 | -0.003 | -0.028 |
|  | m3 |  |  |  |  |  |  | 2 | 0.010 | -0.065 | 0.050 | -0.003 | 0.038 | **0.529** | **0.618** | 0.253 | **0.887** |
|  | m3 |  |  |  |  |  |  | 3 | 0.060 | 0.018 | -0.024 | 0.308 | -0.003 | 0.270 | 0.184 | **0.585** | -0.010 |
| Belgium (Wallonia) | m1 | 2610 | 18 | 0.981 | 0.975 | 0.038 | 0.039 | 1 | **0.737** | **0.709** | **0.735** | **0.460** | **0.600** | **0.664** | **0.701** | **0.569** | **0.748** |
|  | m2 | 2610 | 26 | - | - | - | - | - |  |  |  |  |  |  |  |  |  |
|  | m3 | 2610 | 33 | - | - | - | - | - |  |  |  |  |  |  |  |  |  |
| Canada | m1 | 6177 | 18 | 0.970 | 0.960 | 0.032 | 0.052 | 1 | **0.739** | **0.793** | **0.747** | **0.575** | **0.659** | **0.733** | **0.727** | **0.603** | **0.779** |
|  | m2 | 6177 | 26 | 0.994 | 0.989 | 0.017 | 0.025 | 1 | **0.636** | **0.921** | **0.614** | 0.114 | **0.217** | 0.034 | -0.044 | 0.083 | -0.007 |
|  |  |  |  |  |  |  |  | 2 | **0.177** | -0.007 | **0.212** | **0.501** | **0.495** | **0.739** | **0.812** | **0.560** | **0.830** |
|  | m3 | 6177 | 33 | - | - | - | - | - |  |  |  |  |  |  |  |  |  |
| Croatia | m1 | 2456 | 18 | 0.977 | 0.970 | 0.049 | 0.046 | 1 | **0.723** | **0.770** | **0.753** | **0.559** | **0.713** | **0.760** | **0.746** | **0.690** | **0.763** |
|  | m2 | 2456 | 26 | 0.996 | 0.993 | 0.023 | 0.022 | 1 | **0.754** | **0.865** | **0.795** | 0.140 | **0.245** | 0.126 | -0.003 | **0.177** | -0.052 |
|  |  |  |  |  |  |  |  | 2 | 0.022 | -0.036 | 0.014 | **0.454** | **0.512** | **0.679** | **0.781** | **0.553** | **0.861** |
|  | m3 | 2456 | 33 | - | - | - | - | - |  |  |  |  |  |  |  |  |  |
| Notes: EFA = exploratory factor analysis; the EFA was conducted using the Mplus default settings for EFA with categorical indicators (using Oblique Geomin rotation, Weighted Least Square Means and Variance adjusted estimation, with a probit regression link); Par. = number of free parameters; CFI = comparative fit index; TLI = Tucker Lewis index; RMSEA = root mean square error of approximation; SRMR = standardized root mean square residual; (1) = preoccupation; (2) = tolerance; (3) = withdrawal; (4) = persistence; (5) = displacement; (6) = problem; (7) = deception; (8) = escape; (9) = conflict; Models without results (‘-’) showed estimation problems. Boldface factor loadings denote significant factor loadings at *p* < 0.05; Dark gray cells denote factor loadings > 0.50. | | | | | | | | | | | | | | | | | |

| **Table S4 (continued): EFA factor solutions, calibration samples, by country (n = 111,254 in 44 countries)** | | | | | | | | | | | | | | | | | |
| --- | --- | --- | --- | --- | --- | --- | --- | --- | --- | --- | --- | --- | --- | --- | --- | --- | --- |
|  |  |  | **Model fit** | | | | | **Factor loadings** | | | | | | | | | |
| **Country** | **Model** | ***n*** | **Par.** | **CFI** | **TLI** | **RMSEA** | **SRMR** | **Factor** | **(1)** | **(2)** | **(3)** | **(4)** | **(5)** | **(6)** | **(7)** | **(8)** | **(9)** |
| Czechia | m1 | 5581 | 18 | 0.978 | 0.971 | 0.037 | 0.042 | 1 | **0.752** | **0.799** | **0.745** | **0.573** | **0.687** | **0.664** | **0.736** | **0.645** | **0.724** |
|  | m2 | 5581 | 26 | - | - | - | - | - |  |  |  |  |  |  |  |  |  |
|  | m3 | 5581 | 33 | - | - | - | - | - |  |  |  |  |  |  |  |  |  |
| Denmark | m1 | 1556 | 18 | 0.973 | 0.964 | 0.042 | 0.053 | 1 | **0.669** | **0.803** | **0.775** | **0.514** | **0.694** | **0.718** | **0.763** | **0.548** | **0.859** |
|  | m2 | 1556 | 26 | 0.989 | 0.979 | 0.032 | 0.038 | 1 | **0.399** | **0.904** | **0.775** | 0.016 | 0.113 | -0.159 | 0.030 | 0.201 | 0.002 |
|  |  |  |  |  |  |  |  | 2 | **0.323** | -0.002 | 0.087 | **0.517** | **0.610** | **0.889** | **0.759** | **0.380** | **0.886** |
|  | m3 | 1556 | 33 | - | - | - | - | - |  |  |  |  |  |  |  |  |  |
| England | m1 | 1653 | 18 | 0.979 | 0.972 | 0.047 | 0.045 | 1 | **0.769** | **0.826** | **0.752** | **0.622** | **0.700** | **0.738** | **0.730** | **0.661** | **0.791** |
|  | m2 | 1653 | 26 | 0.996 | 0.993 | 0.023 | 0.026 | 1 | **0.768** | **0.903** | **0.790** | **0.333** | **0.405** | -0.017 | **0.244** | **0.376** | 0.001 |
|  |  |  |  |  |  |  |  | 2 | 0.046 | -0.032 | -0.001 | **0.338** | **0.349** | **0.827** | **0.551** | **0.337** | **0.866** |
|  | m3 | 1653 | 33 | - | - | - | - | - |  |  |  |  |  |  |  |  |  |
| Estonia | m1 | 2311 | 18 | 0.974 | 0.966 | 0.043 | 0.050 | 1 | **0.774** | **0.784** | **0.723** | **0.570** | **0.595** | **0.634** | **0.722** | **0.552** | **0.736** |
|  | m2 | 2311 | 26 | - | - | - | - | - |  |  |  |  |  |  |  |  |  |
|  | m3 | 2311 | 33 | - | - | - | - | - |  |  |  |  |  |  |  |  |  |
| Finland | m1 | 1533 | 18 | 0.988 | 0.984 | 0.044 | 0.040 | 1 | **0.771** | **0.814** | **0.859** | **0.676** | **0.768** | **0.812** | **0.853** | **0.710** | **0.842** |
|  | m2 | 1533 | 26 | - | - | - | - | - |  |  |  |  |  |  |  |  |  |
|  | m3 | 1533 | 33 | - | - | - | - | - |  |  |  |  |  |  |  |  |  |
| France | m1 | 4310 | 18 | 0.982 | 0.976 | 0.032 | 0.036 | 1 | **0.741** | **0.788** | **0.761** | **0.527** | **0.649** | **0.697** | **0.706** | **0.605** | **0.729** |
|  | m2 | 4310 | 26 | - | - | - | - | - |  |  |  |  |  |  |  |  |  |
|  | m3 | 4310 | 33 | - | - | - | - | - |  |  |  |  |  |  |  |  |  |
| Georgia | m1 | 2033 | 18 | 0.977 | 0.969 | 0.031 | 0.041 | 1 | **0.539** | **0.660** | **0.717** | **0.595** | **0.610** | **0.698** | **0.790** | **0.740** | **0.812** |
|  | m2 | 2033 | 26 | 0.991 | 0.984 | 0.023 | 0.029 | 1 | 0.129 | -0.001 | **0.456** | **0.424** | **0.590** | **0.725** | **0.662** | **0.593** | **0.904** |
|  |  |  |  |  |  |  |  | 2 | **0.496** | **0.848** | **0.329** | **0.222** | 0.046 | -0.005 | 0.177 | 0.197 | -0.066 |
|  | m3 | 2033 | 33 | - | - | - | - | - |  |  |  |  |  |  |  |  |  |
| Germany | m1 | 2063 | 18 | 0.976 | 0.968 | 0.037 | 0.042 | 1 | **0.664** | **0.710** | **0.718** | **0.550** | **0.572** | **0.697** | **0.719** | **0.607** | **0.740** |
|  | m2 | 2063 | 26 | - | - | - | - | - |  |  |  |  |  |  |  |  |  |
|  | m3 | 2063 | 33 | - | - | - | - | - |  |  |  |  |  |  |  |  |  |
| Greece | m1 | 1857 | 18 | 0.991 | 0.988 | 0.023 | 0.032 | 1 | **0.729** | **0.674** | **0.660** | **0.440** | **0.475** | **0.679** | **0.655** | **0.593** | **0.734** |
|  | m2 | 1857 | 26 | - | - | - | - | - |  |  |  |  |  |  |  |  |  |
|  | m3 | 1857 | 33 | - | - | - | - | - |  |  |  |  |  |  |  |  |  |
| Hungary | m1 | 1857 | 18 | 0.966 | 0.955 | 0.040 | 0.052 | 1 | **0.741** | **0.687** | **0.633** | **0.465** | **0.578** | **0.604** | **0.651** | **0.574** | **0.695** |
|  | m2 | 1857 | 26 | - | - | - | - | - |  |  |  |  |  |  |  |  |  |
|  | m3 | 1857 | 33 | - | - | - | - | - |  |  |  |  |  |  |  |  |  |
| Notes: EFA = exploratory factor analysis; the EFA was conducted using the Mplus default settings for EFA with categorical indicators (using Oblique Geomin rotation, Weighted Least Square Means and Variance adjusted estimation, with a probit regression link); Par. = number of free parameters; CFI = comparative fit index; TLI = Tucker Lewis index; RMSEA = root mean square error of approximation; SRMR = standardized root mean square residual; (1) = preoccupation; (2) = tolerance; (3) = withdrawal; (4) = persistence; (5) = displacement; (6) = problem; (7) = deception; (8) = escape; (9) = conflict; Models without results (‘-’) showed estimation problems. Boldface factor loadings denote significant factor loadings at *p* < 0.05; Dark gray cells denote factor loadings > 0.50. | | | | | | | | | | | | | | | | | |

| **Table S4 (continued): EFA factor solutions, calibration samples, by country (n = 111,254 in 44 countries)** | | | | | | | | | | | | | | | | | |
| --- | --- | --- | --- | --- | --- | --- | --- | --- | --- | --- | --- | --- | --- | --- | --- | --- | --- |
|  |  |  | **Model fit** | | | | | **Factor loadings** | | | | | | | | | |
| **Country** | **Model** | ***n*** | **Par.** | **CFI** | **TLI** | **RMSEA** | **SRMR** | **Factor** | **(1)** | **(2)** | **(3)** | **(4)** | **(5)** | **(6)** | **(7)** | **(8)** | **(9)** |
| Iceland | m1 | 3346 | 18 | 0.991 | 0.988 | 0.032 | 0.035 | 1 | **0.748** | **0.810** | **0.843** | **0.616** | **0.798** | **0.813** | **0.807** | **0.743** | **0.872** |
|  | m2 | 3346 | 26 | - | - | - | - | - |  |  |  |  |  |  |  |  |  |
|  | m3 | 3346 | 33 | - | - | - | - | - |  |  |  |  |  |  |  |  |  |
| Ireland | m1 | 1814 | 18 | 0.968 | 0.958 | 0.055 | 0.052 | 1 | **0.668** | **0.781** | **0.763** | **0.503** | **0.624** | **0.743** | **0.716** | **0.621** | **0.817** |
|  | m2 | 1814 | 26 | 0.995 | 0.991 | 0.026 | 0.024 | 1 | **0.635** | **0.819** | **0.849** | -0.071 | 0.050 | 0.037 | -0.190 | **0.212** | 0.029 |
|  |  |  |  |  |  |  |  | 2 | 0.089 | 0.032 | -0.014 | **0.591** | **0.598** | **0.734** | **0.934** | **0.443** | **0.824** |
|  | m3 | 1814 | 33 | - | - | - | - | - |  |  |  |  |  |  |  |  |  |
| Israel | m1 | 3567 | 18 | 0.966 | 0.955 | 0.037 | 0.043 | 1 | **0.487** | **0.661** | **0.733** | **0.546** | **0.661** | **0.712** | **0.759** | **0.673** | **0.813** |
|  | m2 | 3567 | 26 | - | - | - | - | - |  |  |  |  |  |  |  |  |  |
|  | m3 | 3567 | 33 | - | - | - | - | - |  |  |  |  |  |  |  |  |  |
| Italy | m1 | 2034 | 18 | 0.981 | 0.974 | 0.034 | 0.040 | 1 | **0.654** | **0.594** | **0.675** | **0.578** | **0.552** | **0.666** | **0.673** | **0.538** | **0.738** |
|  | m2 | 2034 | 26 | - | - | - | - | - |  |  |  |  |  |  |  |  |  |
|  | m3 | 2034 | 33 | - | - | - | - | - |  |  |  |  |  |  |  |  |  |
| Kazakhstan | m1 | 2244 | 18 | 0.979 | 0.973 | 0.035 | 0.041 | 1 | **0.675** | **0.736** | **0.703** | **0.713** | **0.708** | **0.754** | **0.771** | **0.681** | **0.787** |
|  | m2 | 2244 | 26 | 0.994 | 0.989 | 0.022 | 0.026 | 1 | **0.356** | **0.322** | **0.471** | -0.023 | **0.515** | **0.391** | **0.853** | 0.016 | **0.826** |
|  |  |  |  |  |  |  |  | 2 | **0.375** | **0.476** | 0.290 | **0.808** | 0.252 | **0.426** | -0.023 | **0.724** | 0.017 |
|  | m3 | 2244 | 33 | - | - | - | - | - |  |  |  |  |  |  |  |  |  |
| Latvia | m1 | 2071 | 18 | 0.980 | 0.974 | 0.036 | 0.043 | 1 | **0.683** | **0.748** | **0.660** | **0.442** | **0.629** | **0.768** | **0.748** | **0.507** | **0.725** |
|  | m2 | 2071 | 26 | 0.991 | 0.983 | 0.029 | 0.030 | 1 | -0.002 | **0.334** | **0.342** | **0.314** | **0.413** | **0.914** | **0.656** | **0.324** | **0.757** |
|  |  |  |  |  |  |  |  | 2 | **-0.823** | **-0.504** | **-0.392** | -0.169 | **-0.277** | 0.107 | -0.141 | **-0.233** | -0.006 |
|  | m3 | 2071 | 33 | - | - | - | - | - |  |  |  |  |  |  |  |  |  |
| Lithuania | m1 | 1842 | 18 | 0.974 | 0.966 | 0.045 | 0.047 | 1 | **0.786** | **0.771** | **0.786** | **0.615** | **0.704** | **0.709** | **0.792** | **0.603** | **0.784** |
|  | m2 | 1842 | 26 | - | - | - | - | - |  |  |  |  |  |  |  |  |  |
|  | m3 | 1842 | 33 | - | - | - | - | - |  |  |  |  |  |  |  |  |  |
| Luxembourg | m1 | 1944 | 18 | 0.980 | 0.973 | 0.033 | 0.042 | 1 | **0.721** | **0.678** | **0.657** | **0.507** | **0.570** | **0.660** | **0.622** | **0.510** | **0.682** |
|  | m2 | 1944 | 26 | - | - | - | - | - |  |  |  |  |  |  |  |  |  |
|  | m3 | 1944 | 33 | - | - | - | - | - |  |  |  |  |  |  |  |  |  |
| Malta | m1 | 1252 | 18 | 0.991 | 0.988 | 0.023 | 0.035 | 1 | **0.732** | **0.661** | **0.709** | **0.544** | **0.565** | **0.662** | **0.652** | **0.566** | **0.672** |
|  | m2 | 1252 | 26 | - | - | - | - | - |  |  |  |  |  |  |  |  |  |
|  | m3 | 1252 | 33 | - | - | - | - | - |  |  |  |  |  |  |  |  |  |
| Netherlands | m1 | 2289 | 18 | 0.992 | 0.989 | 0.019 | 0.034 | 1 | **0.487** | **0.728** | **0.702** | **0.550** | **0.579** | **0.717** | **0.727** | **0.578** | **0.780** |
|  | m2 | 2289 | 26 | 0.997 | 0.994 | 0.013 | 0.025 | 1 | **0.362** | **0.674** | **0.790** | 0.126 | 0.176 | -0.004 | -0.033 | 0.237 | 0.140 |
|  |  |  |  |  |  |  |  | 2 | 0.161 | 0.110 | -0.006 | **0.450** | **0.432** | **0.750** | **0.789** | **0.372** | **0.672** |
|  | m3 | 2289 | 33 | - | - | - | - | - |  |  |  |  |  |  |  |  |  |
| Notes: EFA = exploratory factor analysis; the EFA was conducted using the Mplus default settings for EFA with categorical indicators (using Oblique Geomin rotation, Weighted Least Square Means and Variance adjusted estimation, with a probit regression link); Par. = number of free parameters; CFI = comparative fit index; TLI = Tucker Lewis index; RMSEA = root mean square error of approximation; SRMR = standardized root mean square residual; (1) = preoccupation; (2) = tolerance; (3) = withdrawal; (4) = persistence; (5) = displacement; (6) = problem; (7) = deception; (8) = escape; (9) = conflict; Models without results (‘-’) showed estimation problems. Boldface factor loadings denote significant factor loadings at *p* < 0.05; Dark gray cells denote factor loadings > 0.50. | | | | | | | | | | | | | | | | | |

| **Table S4 (continued): EFA factor solutions, calibration samples, by country (n = 111,254 in 44 countries)** | | | | | | | | | | | | | | | | | |
| --- | --- | --- | --- | --- | --- | --- | --- | --- | --- | --- | --- | --- | --- | --- | --- | --- | --- |
|  |  |  | **Model fit** | | | | | **Factor loadings** | | | | | | | | | |
| **Country** | **Model** | ***n*** | **Par.** | **CFI** | **TLI** | **RMSEA** | **SRMR** | **Factor** | **(1)** | **(2)** | **(3)** | **(4)** | **(5)** | **(6)** | **(7)** | **(8)** | **(9)** |
| North Macedonia | m1 | 2287 | 18 | 0.979 | 0.972 | 0.038 | 0.041 | 1 | **0.712** | **0.734** | **0.635** | **0.443** | **0.528** | **0.682** | **0.762** | **0.524** | **0.783** |
|  | m2 | 2287 | 26 | 0.992 | 0.985 | 0.028 | 0.028 | 1 | **0.762** | **0.787** | **0.599** | **0.309** | 0.144 | -0.108 | 0.091 | **0.471** | -0.002 |
|  |  |  |  |  |  |  |  | 2 | -0.008 | -0.017 | 0.063 | 0.154 | **0.411** | **0.829** | **0.709** | 0.075 | **0.832** |
|  | m3 | 2287 | 33 | - | - | - | - | - |  |  |  |  |  |  |  |  |  |
| Norway | m1 | 1526 | 18 | 0.984 | 0.978 | 0.039 | 0.034 | 1 | **0.774** | **0.791** | **0.756** | **0.602** | **0.790** | **0.784** | **0.836** | **0.621** | **0.934** |
|  | m2 | 1526 | 26 | - | - | - | - | - |  |  |  |  |  |  |  |  |  |
|  | m3 | 1526 | 33 | - | - | - | - | - |  |  |  |  |  |  |  |  |  |
| Poland | m1 | 2527 | 18 | 0.980 | 0.973 | 0.038 | 0.045 | 1 | **0.728** | **0.682** | **0.672** | **0.593** | **0.635** | **0.676** | **0.696** | **0.584** | **0.698** |
|  | m2 | 2527 | 26 | 0.992 | 0.985 | 0.028 | 0.029 | 1 | **0.724** | **0.802** | **0.712** | 0.213 | 0.014 | 0.004 | 0.041 | **0.332** | 0.005 |
|  |  |  |  |  |  |  |  | 2 | 0.049 | -0.073 | 0.009 | **0.411** | **0.652** | **0.710** | **0.692** | **0.283** | **0.730** |
|  | m3 | 2527 | 33 | - | - | - | - | - |  |  |  |  |  |  |  |  |  |
| Portugal | m1 | 2933 | 18 | 0.973 | 0.964 | 0.048 | 0.047 | 1 | **0.741** | **0.785** | **0.773** | **0.651** | **0.730** | **0.747** | **0.773** | **0.676** | **0.804** |
|  | m2 | 2933 | 26 | - | - | - | - | - |  |  |  |  |  |  |  |  |  |
|  | m3 | 2933 | 33 | - | - | - | - | - |  |  |  |  |  |  |  |  |  |
| Republic of Moldova | m1 | 2214 | 18 | 0.986 | 0.981 | 0.025 | 0.034 | 1 | **0.635** | **0.603** | **0.644** | **0.477** | **0.594** | **0.535** | **0.685** | **0.491** | **0.683** |
|  | m2 | 2214 | 26 | - | - | - | - | - |  |  |  |  |  |  |  |  |  |
|  | m3 | 2214 | 33 | - | - | - | - | - |  |  |  |  |  |  |  |  |  |
| Romania | m1 | 2241 | 18 | 0.982 | 0.976 | 0.032 | 0.038 | 1 | **0.573** | **0.664** | **0.678** | **0.418** | **0.607** | **0.667** | **0.706** | **0.553** | **0.715** |
|  | m2 | 2241 | 26 | - | - | - | - | - |  |  |  |  |  |  |  |  |  |
|  | m3 | 2241 | 33 | - | - | - | - | - |  |  |  |  |  |  |  |  |  |
| Russian Federation | m1 | 2030 | 18 | 0.990 | 0.986 | 0.033 | 0.034 | 1 | **0.757** | **0.803** | **0.767** | **0.708** | **0.692** | **0.759** | **0.845** | **0.709** | **0.785** |
|  | m2 | 2030 | 26 | - | - | - | - | - |  |  |  |  |  |  |  |  |  |
|  | m3 | 2030 | 33 | - | - | - | - | - |  |  |  |  |  |  |  |  |  |
| Scotland | m1 | 2458 | 18 | 0.972 | 0.963 | 0.045 | 0.054 | 1 | **0.699** | **0.802** | **0.752** | **0.580** | **0.707** | **0.739** | **0.733** | **0.647** | **0.788** |
|  | m2 | 2458 | 26 | 0.997 | 0.995 | 0.017 | 0.025 | 1 | **0.612** | **0.959** | **0.538** | 0.077 | **0.241** | 0.060 | -0.032 | **0.195** | -0.014 |
|  |  |  |  |  |  |  |  | 2 | **0.174** | -0.004 | **0.297** | **0.555** | **0.531** | **0.733** | **0.805** | **0.511** | **0.844** |
|  | m3 | 2458 | 33 | - | - | - | - | - |  |  |  |  |  |  |  |  |  |
| Serbia | m1 | 1870 | 18 | 0.973 | 0.963 | 0.050 | 0.056 | 1 | **0.722** | **0.782** | **0.806** | **0.521** | **0.733** | **0.763** | **0.745** | **0.615** | **0.795** |
|  | m2 | 1870 | 26 | 0.990 | 0.981 | 0.036 | 0.034 | 1 | **0.578** | **0.736** | **0.916** | 0.030 | **0.258** | 0.024 | -0.015 | 0.058 | -0.001 |
|  |  |  |  |  |  |  |  | 2 | **0.214** | 0.115 | -0.011 | **0.516** | **0.525** | **0.779** | **0.793** | **0.592** | **0.835** |
|  | m3 | 1870 | 33 | 0.997 | 0.990 | 0.026 | 0.020 | 1 | **0.552** | **0.695** | **0.817** | 0.006 | **0.252** | 0.009 | 0.024 | -0.014 | -0.030 |
| Slovenia | m1 | 2563 | 18 | 0.990 | 0.986 | 0.029 | 0.033 | 1 | **0.752** | **0.802** | **0.775** | **0.488** | **0.704** | **0.775** | **0.757** | **0.694** | **0.794** |
|  | m2 | 2563 | 26 | - | - | - | - | - |  |  |  |  |  |  |  |  |  |
|  | m3 | 2563 | 33 | - | - | - | - | - |  |  |  |  |  |  |  |  |  |
| Notes: EFA = exploratory factor analysis; the EFA was conducted using the Mplus default settings for EFA with categorical indicators (using Oblique Geomin rotation, Weighted Least Square Means and Variance adjusted estimation, with a probit regression link); Par. = number of free parameters; CFI = comparative fit index; TLI = Tucker Lewis index; RMSEA = root mean square error of approximation; SRMR = standardized root mean square residual; (1) = preoccupation; (2) = tolerance; (3) = withdrawal; (4) = persistence; (5) = displacement; (6) = problem; (7) = deception; (8) = escape; (9) = conflict; Models without results (‘-’) showed estimation problems. Boldface factor loadings denote significant factor loadings at *p* < 0.05; Dark gray cells denote factor loadings > 0.50. | | | | | | | | | | | | | | | | | |

| **Table S4 (continued): EFA factor solutions, calibration samples, by country (n = 111,254 in 44 countries)** | | | | | | | | | | | | | | | | | |
| --- | --- | --- | --- | --- | --- | --- | --- | --- | --- | --- | --- | --- | --- | --- | --- | --- | --- |
|  |  |  | **Model fit** | | | | | **Factor loadings** | | | | | | | | | |
| **Country** | **Model** | ***n*** | **Par.** | **CFI** | **TLI** | **RMSEA** | **SRMR** | **Factor** | **(1)** | **(2)** | **(3)** | **(4)** | **(5)** | **(6)** | **(7)** | **(8)** | **(9)** |
| Spain | m1 | 2035 | 18 | 0.984 | 0.978 | 0.047 | 0.043 | 1 | **0.766** | **0.741** | **0.773** | **0.666** | **0.684** | **0.726** | **0.784** | **0.700** | **0.801** |
|  | m2 | 2035 | 26 | - | - | - | - | - |  |  |  |  |  |  |  |  |  |
|  | m3 | 2035 | 33 | - | - | - | - | - |  |  |  |  |  |  |  |  |  |
| Sweden | m1 | 2003 | 18 | 0.964 | 0.952 | 0.048 | 0.062 | 1 | **0.653** | **0.662** | **0.749** | **0.632** | **0.608** | **0.727** | **0.757** | **0.619** | **0.742** |
|  | m2 | 2003 | 26 | - | - | - | - | - |  |  |  |  |  |  |  |  |  |
|  | m3 | 2003 | 33 | - | - | - | - | - |  |  |  |  |  |  |  |  |  |
| Switzerland | m1 | 3561 | 18 | 0.988 | 0.984 | 0.023 | 0.030 | 1 | **0.705** | **0.712** | **0.635** | **0.476** | **0.520** | **0.647** | **0.600** | **0.554** | **0.658** |
|  | m2 | 3561 | 26 | - | - | - | - | - |  |  |  |  |  |  |  |  |  |
|  | m3 | 3561 | 33 | - | - | - | - | - |  |  |  |  |  |  |  |  |  |
| Turkey | m1 | 2770 | 18 | 0.987 | 0.982 | 0.032 | 0.035 | 1 | **0.682** | **0.623** | **0.661** | **0.630** | **0.636** | **0.740** | **0.725** | **0.625** | **0.750** |
|  | m2 | 2770 | 26 | 0.995 | 0.990 | 0.024 | 0.023 | 1 | **0.426** | -0.007 | 0.227 | **0.309** | **0.697** | **0.710** | **0.700** | 0.154 | **0.789** |
|  |  |  |  |  |  |  |  | 2 | **0.301** | **0.714** | **0.495** | **0.366** | -0.041 | 0.060 | 0.054 | **0.531** | -0.010 |
|  | m3 | 2770 | 33 | 0.999 | 0.997 | 0.013 | 0.013 | 1 | **0.363** | -0.082 | 0.010 | **0.249** | **0.671** | **0.807** | **0.702** | -0.002 | **0.802** |
|  |  |  |  |  |  |  |  | 2 | **0.313** | **0.656** | **0.626** | 0.351 | -0.009 | -0.031 | 0.054 | 0.556 | -0.015 |
|  |  |  |  |  |  |  |  | 3 | 0.042 | 0.120 | -0.033 | 0.217 | -0.043 | 0.178 | -0.160 | 0.399 | 0.004 |
| Ukraine | m1 | 3116 | 18 | 0.972 | 0.963 | 0.044 | 0.049 | 1 | **0.687** | **0.756** | **0.704** | **0.462** | **0.731** | **0.741** | **0.766** | **0.506** | **0.755** |
|  | m2 | 3116 | 26 | - | - | - | - | - |  |  |  |  |  |  |  |  |  |
|  | m3 | 3116 | 33 | - | - | - | - | - |  |  |  |  |  |  |  |  |  |
| Wales | m1 | 7728 | 18 | 0.980 | 0.974 | 0.048 | 0.039 | 1 | **0.767** | **0.827** | **0.792** | **0.655** | **0.740** | **0.744** | **0.785** | **0.658** | **0.815** |
|  | m2 | 7728 | 26 | 0.997 | 0.995 | 0.022 | 0.018 | 1 | **0.771** | **0.917** | **0.700** | **0.284** | **0.292** | 0.002 | 0.064 | **0.244** | -0.059 |
|  |  |  |  |  |  |  |  | 2 | 0.043 | -0.033 | 0.143 | **0.409** | **0.490** | **0.785** | **0.763** | **0.452** | **0.912** |
|  | m3 | 7728 | 33 | - | - | - | - | - |  |  |  |  |  |  |  |  |  |
| Notes: EFA = exploratory factor analysis; the EFA was conducted using the Mplus default settings for EFA with categorical indicators (using Oblique Geomin rotation, Weighted Least Square Means and Variance adjusted estimation, with a probit regression link); Par. = number of free parameters; CFI = comparative fit index; TLI = Tucker Lewis index; RMSEA = root mean square error of approximation; SRMR = standardized root mean square residual; (1) = preoccupation; (2) = tolerance; (3) = withdrawal; (4) = persistence; (5) = displacement; (6) = problem; (7) = deception; (8) = escape; (9) = conflict; Models without results (‘-’) showed estimation problems. Boldface factor loadings denote significant factor loadings at *p* < 0.05; Dark gray cells denote factor loadings > 0.50. | | | | | | | | | | | | | | | | | |

**Summary EFA results (Tables S3 and S4)**

We evaluated the EFAs based on empirical eigenvalues and parallel analysis using the calibration samples for each country. The number of factors with empirical eigenvalues higher than one denotes the number of potential factors to retain. We compared the empirical eigenvalues values with 1,000 randomly generated eigenvalues, based on the same number of items and sample size of the respective country. The number of factors to retain was determined by the number of factors where the 95^th^ percentile random data eigenvalues did not exceed the empirical eigenvalues[4]. In 42 out of 44 countries, results from the EFA identified one factor with an eigenvalue higher than one, suggesting a one-factor solution in these countries (Table S3). For Sweden and Ukraine, two factors showed an eigenvalue higher than one, suggesting a two-factor solution. However, the parallel analysis did not replicate this finding, because the empirical eigenvalue of only the first factor exceeded its 95^th^ random eigenvalue. Thus, also in these two countries, a one-factor solution was supported.

Nevertheless, for each country, we estimated the model estimates from the one-, two-, and three-factor solutions. Model fit was evaluated based on the Comparative Fit Index (CFI), Tucker Lewis Index (TLI), Root Mean Square Error of Approximation (RMSEA), and Standardized Root Mean Square Residual (SRMR) (CFI/TLI: ≥ 0.90 acceptable, ≥ 0.95 good; RMSEA: ≤ 0.08 acceptable, ≤ 0.06 good; SRMR: ≤ 0.10 acceptable, ≤ 0.08 good)[5]. We did not rely on the Chi-square statistic given its sensitivity to large sample sizes[6].

In all 44 countries, the model fit of the one-factor solution was good according to all fit indices, because the lowest observed CFI and TLI were 0.964 and 0.952 and the highest RMSEA and SRMR 0.055 and 0.062, respectively (Table S4). We also evaluated the quality of the one-factor solution, whereby the quality of the factor was considered good when there were at least five items with significant (*p* < 0.05) factor loadings higher than 0.50[7]. In all countries, this requirement was fulfilled (Table S4). More specifically, in 30 out of 44 countries, all nine factor loadings exceeded 0.50. In 12 countries, there was one factor loading lower than 0.50 (although not lower than 0.42), and in two countries, there were two factor loadings lower than 0.50 (although not lower than 0.44). Thus, in all countries, the model fit and quality of the one-factor model was good.

For 28 out of 44 countries, the two-factor solution yielded estimation problems. Often, these problems emerged because the two factors showed correlations equal or greater than one. Such model estimates should not be interpreted and warrant re-specification[8]. Hence, for these countries, the two-factor solutions were considered inappropriate. From the 16 countries that showed no estimation problems, the two-factor model showed better model fit than the one-factor model (Table S4). Subsequently, we evaluated the quality of the two-factor solutions, whereby each factor should consist of at least three items with significant (*p* < 0.05) factor loadings higher than 0.50 without any cross-loadings that differed less than 0.20[7,9]. Six out of the 16 countries without estimation problems did not meet this requirement (Denmark, Georgia, Kazakhstan, Latvia, Netherlands, and Turkey), suggesting the quality of the two-factor solution was poor in these countries. Nine out of the 16 countries showed one factor with the items preoccupation, tolerance, and withdrawal and a second factor that was not consistent across countries (Canada, Croatia, England, Ireland, North Macedonia, Poland, Scotland, Serbia, and Wales). More specifically, after removal of items with factor loadings < 0.50 and cross-loadings, the second factor consisted of at least three of the items persistence, displacement, problem, deception, escape, and conflict. One out of the 16 countries showed one factor with items preoccupation, tolerance, withdrawal, and displacement and a second factor with items problems, deception, and conflict (Belgium: Flanders).

For 41 out of 44 countries, the three-factor solution yielded estimation problems, also mostly because factor correlations were equal or greater than one. Hence, the three-factor solution was considered inappropriate for these countries[8]. From the three countries that showed no estimation problems, the three-factor model showed better model fit than the one- and two-factor solution (Austria, Belgium: Flanders, and Turkey). However, the quality of the three-factor solutions was poor, because after removal of items with factor loadings < 0.50 and cross-loadings, the second and/or third factor consisted of less than three items (Table S4).

In sum, in 34 out of 44 countries, results from the eigenvalues and parallel analysis suggested a one-factor solution and, accordingly, the quality of the two- and three-factor solutions was poor. In the 10 other countries, the quality of the two-factor solution was acceptable, however, the eigenvalues and parallel analysis suggested a one-factor solution. As such, there was insufficient evidence for two-factor models. Furthermore, the model fit of the one-factor model was good in all countries, as well as the quality of the factor. Thus, we consider the factor structure as unidimensional.

| **Table S5: CFA model fit and reliability, validation samples, by country (n = 111,278 in 44 countries)** | | | | | | | | |
| --- | --- | --- | --- | --- | --- | --- | --- | --- |
| **Country** | ***n*** | **CFI** | **TLI** | **RMSEA** | **SRMR** | **Min. loading^1^** | **Max. loading^2^** | **Internal consistency^3^** |
| Albania | 825 | 0.988 | 0.984 | 0.029 | 0.040 | 0.470 | 0.763 | 0.867 |
| Armenia | 2215 | 0.994 | 0.991 | 0.022 | 0.030 | 0.606 | 0.765 | 0.900 |
| Austria | 2006 | 0.977 | 0.969 | 0.037 | 0.046 | 0.544 | 0.729 | 0.864 |
| Azerbaijan | 1889 | 0.980 | 0.973 | 0.040 | 0.039 | 0.754 | 0.874 | 0.951 |
| Belgium (Flanders) | 2059 | 0.976 | 0.968 | 0.039 | 0.044 | 0.501 | 0.774 | 0.872 |
| Belgium (Wallonia) | 2611 | 0.984 | 0.978 | 0.035 | 0.036 | 0.486 | 0.761 | 0.871 |
| Canada | 6178 | 0.975 | 0.967 | 0.031 | 0.046 | 0.598 | 0.834 | 0.907 |
| Croatia | 2457 | 0.978 | 0.970 | 0.047 | 0.042 | 0.602 | 0.828 | 0.911 |
| Czechia | 5581 | 0.979 | 0.972 | 0.038 | 0.043 | 0.560 | 0.799 | 0.899 |
| Denmark | 1557 | 0.973 | 0.965 | 0.044 | 0.057 | 0.568 | 0.806 | 0.900 |
| England | 1653 | 0.972 | 0.963 | 0.043 | 0.056 | 0.588 | 0.830 | 0.892 |
| Estonia | 2311 | 0.977 | 0.969 | 0.043 | 0.047 | 0.577 | 0.810 | 0.900 |
| Finland | 1534 | 0.982 | 0.976 | 0.044 | 0.044 | 0.664 | 0.857 | 0.924 |
| France | 4311 | 0.976 | 0.968 | 0.036 | 0.042 | 0.519 | 0.803 | 0.887 |
| Georgia | 2034 | 0.990 | 0.986 | 0.022 | 0.038 | 0.540 | 0.765 | 0.878 |
| Germany | 2063 | 0.986 | 0.981 | 0.028 | 0.037 | 0.544 | 0.713 | 0.867 |
| Greece | 1858 | 0.981 | 0.975 | 0.032 | 0.038 | 0.380 | 0.759 | 0.840 |
| Hungary | 1858 | 0.963 | 0.951 | 0.043 | 0.051 | 0.401 | 0.703 | 0.844 |
| Iceland | 3347 | 0.988 | 0.984 | 0.031 | 0.036 | 0.603 | 0.858 | 0.927 |
| Ireland | 1814 | 0.966 | 0.955 | 0.057 | 0.053 | 0.554 | 0.791 | 0.886 |
| Israel | 3567 | 0.979 | 0.972 | 0.032 | 0.037 | 0.527 | 0.832 | 0.889 |
| Italy | 2035 | 0.978 | 0.971 | 0.037 | 0.041 | 0.516 | 0.716 | 0.852 |
| Kazakhstan | 2244 | 0.986 | 0.981 | 0.030 | 0.039 | 0.632 | 0.810 | 0.913 |
| Latvia | 2072 | 0.984 | 0.978 | 0.033 | 0.041 | 0.428 | 0.801 | 0.872 |
| Lithuania | 1843 | 0.983 | 0.978 | 0.041 | 0.042 | 0.593 | 0.813 | 0.914 |
| Luxembourg | 1945 | 0.975 | 0.967 | 0.039 | 0.044 | 0.522 | 0.742 | 0.856 |
| Malta | 2288 | 0.977 | 0.969 | 0.038 | 0.042 | 0.451 | 0.775 | 0.859 |
| Netherlands | 1252 | 0.979 | 0.972 | 0.040 | 0.047 | 0.506 | 0.768 | 0.858 |
| North Macedonia | 2290 | 0.988 | 0.984 | 0.023 | 0.037 | 0.524 | 0.744 | 0.875 |
| Norway | 1527 | 0.987 | 0.983 | 0.035 | 0.032 | 0.603 | 0.930 | 0.934 |
| Poland | 2528 | 0.979 | 0.972 | 0.041 | 0.042 | 0.574 | 0.798 | 0.890 |
| Portugal | 2933 | 0.981 | 0.974 | 0.041 | 0.041 | 0.610 | 0.788 | 0.908 |
| Republic of Moldova | 2215 | 0.987 | 0.982 | 0.025 | 0.033 | 0.470 | 0.716 | 0.841 |
| Romania | 2242 | 0.987 | 0.983 | 0.028 | 0.031 | 0.430 | 0.712 | 0.848 |
| Russian Federation | 2031 | 0.990 | 0.986 | 0.035 | 0.032 | 0.694 | 0.826 | 0.927 |
| Scotland | 2458 | 0.968 | 0.958 | 0.051 | 0.060 | 0.561 | 0.837 | 0.900 |
| Serbia | 1870 | 0.983 | 0.978 | 0.042 | 0.042 | 0.603 | 0.804 | 0.910 |
| Slovenia | 2563 | 0.990 | 0.986 | 0.029 | 0.036 | 0.492 | 0.790 | 0.902 |
| Spain | 2035 | 0.986 | 0.981 | 0.040 | 0.036 | 0.604 | 0.809 | 0.906 |
| Sweden | 2003 | 0.978 | 0.970 | 0.045 | 0.048 | 0.603 | 0.793 | 0.896 |
| Switzerland | 3561 | 0.971 | 0.962 | 0.037 | 0.044 | 0.494 | 0.741 | 0.844 |
| Turkey | 2771 | 0.981 | 0.975 | 0.039 | 0.039 | 0.614 | 0.750 | 0.883 |
| Ukraine | 3116 | 0.979 | 0.972 | 0.038 | 0.042 | 0.481 | 0.786 | 0.885 |
| Wales | 7728 | 0.980 | 0.973 | 0.048 | 0.040 | 0.649 | 0.840 | 0.921 |
| Notes: CFA = confirmatory factor analysis; SMU = social media use; CFI = comparative fit index; TLI = Tucker Lewis index; RMSEA = root mean square error of approximation; SRMR = standardized root mean square residual.  ^1^ Lowest observed factor loading from nine items.  ^2^ Highest observed factor loading from nine items.  ^3^ Based on ordinal alpha. | | | | | | | | |

| **Table S6: CFA factor loadings problematic SMU, validation samples, by country (n = 111,278 in 44 countries)** | | | | | | | | | | |
| --- | --- | --- | --- | --- | --- | --- | --- | --- | --- | --- |
|  |  | **Problematic SMU items** | | | | | | | | |
|  | ***n*** | **(1)** | **(2)** | **(3)** | **(4)** | **(5)** | **(6)** | **(7)** | **(8)** | **(9)** |
| Albania | 825 | 0.661 | 0.695 | 0.707 | 0.612 | 0.626 | *0.470* | **0.763** | 0.657 | 0.708 |
| Armenia | 2215 | 0.702 | 0.736 | 0.675 | *0.606* | 0.739 | 0.756 | **0.765** | 0.649 | 0.760 |
| Austria | 2006 | 0.725 | 0.675 | 0.695 | 0.565 | 0.567 | 0.650 | 0.700 | *0.544* | **0.729** |
| Azerbaijan | 1889 | *0.754* | 0.814 | 0.828 | 0.814 | 0.838 | 0.873 | 0.859 | 0.829 | **0.874** |
| Belgium (Flanders) | 2059 | 0.710 | **0.774** | 0.733 | *0.501* | 0.589 | 0.640 | 0.676 | 0.597 | 0.736 |
| Belgium (Wallonia) | 2611 | 0.660 | 0.733 | 0.721 | *0.486* | 0.589 | 0.666 | 0.720 | 0.582 | **0.761** |
| Canada | 6178 | 0.781 | **0.834** | 0.771 | 0.645 | 0.638 | 0.766 | 0.731 | *0.598* | 0.799 |
| Croatia | 2457 | 0.743 | 0.763 | 0.748 | *0.602* | 0.708 | 0.760 | 0.778 | 0.681 | **0.828** |
| Czechia | 5581 | 0.737 | **0.799** | 0.762 | *0.560* | 0.692 | 0.696 | 0.747 | 0.653 | 0.737 |
| Denmark | 1557 | 0.733 | 0.773 | 0.767 | 0.615 | 0.695 | 0.721 | 0.768 | *0.568* | **0.806** |
| England | 1653 | 0.671 | 0.760 | 0.735 | *0.588* | 0.620 | 0.735 | 0.722 | 0.641 | **0.830** |
| Estonia | 2311 | 0.795 | **0.810** | 0.708 | *0.577* | 0.668 | 0.758 | 0.758 | 0.595 | 0.760 |
| Finland | 1534 | 0.774 | **0.857** | 0.851 | 0.667 | 0.695 | 0.779 | 0.803 | *0.664* | 0.781 |
| France | 4311 | 0.737 | **0.803** | 0.738 | *0.519* | 0.643 | 0.696 | 0.678 | 0.589 | 0.772 |
| Georgia | 2034 | *0.540* | 0.631 | 0.711 | 0.593 | 0.601 | 0.703 | **0.765** | 0.725 | 0.760 |
| Germany | 2063 | 0.704 | 0.688 | 0.707 | *0.544* | 0.587 | 0.654 | **0.713** | 0.585 | 0.686 |
| Greece | 1858 | 0.659 | 0.637 | 0.604 | *0.380* | 0.523 | 0.648 | 0.689 | 0.608 | **0.759** |
| Hungary | 1858 | **0.703** | 0.673 | 0.682 | *0.401* | 0.619 | 0.627 | 0.617 | 0.571 | 0.694 |
| Iceland | 3347 | 0.805 | 0.813 | 0.817 | *0.603* | 0.770 | 0.770 | 0.786 | 0.716 | **0.858** |
| Ireland | 1814 | 0.682 | 0.761 | 0.744 | 0.585 | 0.632 | 0.715 | 0.725 | *0.554* | **0.791** |
| Israel | 3567 | *0.527* | 0.644 | 0.722 | 0.562 | 0.670 | 0.756 | 0.801 | 0.700 | **0.832** |
| Italy | 2035 | **0.716** | 0.630 | 0.695 | 0.551 | 0.546 | 0.635 | 0.654 | *0.516* | 0.713 |
| Kazakhstan | 2244 | *0.632* | 0.771 | 0.747 | 0.678 | 0.698 | 0.804 | 0.795 | 0.701 | **0.810** |
| Latvia | 2072 | 0.675 | 0.729 | 0.657 | *0.428* | 0.635 | **0.801** | 0.753 | 0.500 | 0.779 |
| Lithuania | 1843 | 0.798 | **0.813** | 0.792 | *0.593* | 0.720 | 0.752 | 0.809 | 0.629 | 0.758 |
| Luxembourg | 1945 | **0.742** | 0.682 | 0.659 | 0.539 | *0.522* | 0.687 | 0.654 | *0.522* | 0.709 |
| Malta | 2288 | 0.712 | 0.704 | 0.650 | *0.451* | 0.567 | 0.661 | **0.775** | 0.500 | 0.744 |
| Netherlands | 1252 | 0.671 | **0.768** | 0.742 | 0.508 | 0.614 | 0.656 | 0.641 | *0.506* | 0.617 |
| North Macedonia | 2290 | *0.524* | 0.736 | 0.725 | 0.596 | 0.628 | **0.744** | 0.719 | 0.579 | 0.738 |
| Norway | 1527 | 0.778 | 0.834 | 0.796 | *0.603* | 0.782 | 0.832 | 0.816 | 0.698 | **0.930** |
| Poland | 2528 | **0.798** | 0.735 | 0.747 | *0.574* | 0.673 | 0.704 | 0.701 | 0.575 | 0.727 |
| Portugal | 2933 | 0.734 | **0.788** | 0.780 | *0.610* | 0.732 | 0.743 | 0.759 | 0.629 | 0.781 |
| Republic of Moldova | 2215 | 0.636 | 0.633 | 0.653 | *0.470* | 0.586 | 0.602 | **0.716** | 0.497 | 0.712 |
| Romania | 2242 | 0.599 | 0.634 | 0.671 | *0.430* | 0.589 | 0.670 | 0.682 | 0.603 | **0.712** |
| Russian Federation | 2031 | 0.777 | **0.826** | 0.806 | 0.695 | 0.774 | 0.781 | 0.805 | *0.694* | 0.763 |
| Scotland | 2458 | 0.760 | **0.837** | 0.751 | 0.593 | 0.720 | 0.729 | 0.720 | *0.561* | 0.775 |
| Serbia | 1870 | 0.747 | 0.744 | 0.778 | *0.603* | 0.724 | 0.768 | 0.793 | 0.634 | **0.804** |
| Slovenia | 2563 | 0.758 | 0.770 | 0.746 | *0.492* | 0.671 | 0.757 | 0.761 | 0.690 | **0.790** |
| Spain | 2035 | 0.767 | 0.683 | 0.777 | *0.604* | 0.691 | 0.725 | 0.792 | 0.655 | **0.809** |
| Sweden | 2003 | 0.710 | 0.710 | 0.743 | *0.603* | 0.606 | 0.778 | 0.735 | 0.672 | **0.793** |
| Switzerland | 3561 | **0.741** | 0.683 | 0.686 | *0.494* | 0.509 | 0.701 | 0.589 | 0.496 | 0.659 |
| Turkey | 2771 | 0.647 | 0.668 | 0.689 | *0.614* | 0.655 | **0.750** | 0.718 | 0.636 | 0.736 |
| Ukraine | 3116 | 0.690 | **0.786** | 0.737 | *0.481* | 0.668 | 0.708 | 0.757 | 0.576 | 0.755 |
| Wales | 7728 | 0.768 | **0.840** | 0.790 | *0.649* | 0.748 | 0.753 | 0.775 | 0.676 | 0.811 |
| Notes: SMU = social media use; (1) = preoccupation; (2) = tolerance; (3) = withdrawal; (4) = persistence; (5) = displacement; (6) = problem; (7) = deception; (8) = escape; (9) = conflict. Rates in italics indicate the row minimum with respect to the nine items; rates in boldface indicate the row maximum with respect to the nine items. | | | | | | | | | | |

| **Table S7: Gender measurement invariance, by country (n = 222,532 in 44 countries)** | | | | | | | | | | |
| --- | --- | --- | --- | --- | --- | --- | --- | --- | --- | --- |
|  | **Configural invariance^1^ (par. = 36)** | | | | **Scalar invariance^2^ (par. = 29)** | | | | **Change** | |
|  | **CFI** | **TLI** | **RMSEA** | **SRMR** | **CFI** | **TLI** | **RMSEA** | **SRMR** | **ΔCFI** | **ΔRMSEA** |
| Albania | 0.981 | 0.975 | 0.036 | 0.049 | 0.981 | 0.977 | 0.035 | 0.050 | 0.000 | -0.001 |
| Armenia | 0.989 | 0.985 | 0.030 | 0.039 | 0.987 | 0.984 | 0.030 | 0.040 | -0.002 | 0.000 |
| Austria | 0.982 | 0.977 | 0.030 | 0.039 | 0.978 | 0.975 | 0.032 | 0.042 | -0.004 | 0.002 |
| Azerbaijan | 0.977 | 0.970 | 0.044 | 0.046 | 0.977 | 0.972 | 0.042 | 0.046 | 0.000 | -0.002 |
| Belgium (Flanders) | 0.980 | 0.973 | 0.037 | 0.043 | 0.975 | 0.971 | 0.039 | 0.046 | -0.005 | 0.002 |
| Belgium (Wallonia) | 0.982 | 0.976 | 0.037 | 0.038 | 0.981 | 0.977 | 0.036 | 0.039 | -0.001 | -0.001 |
| Canada | 0.971 | 0.962 | 0.032 | 0.049 | 0.973 | 0.968 | 0.029 | 0.049 | 0.002 | -0.003 |
| Croatia | 0.977 | 0.969 | 0.049 | 0.045 | 0.976 | 0.971 | 0.048 | 0.046 | -0.001 | -0.001 |
| Czechia | 0.978 | 0.970 | 0.038 | 0.043 | 0.976 | 0.972 | 0.037 | 0.045 | -0.002 | -0.001 |
| Denmark | 0.978 | 0.970 | 0.040 | 0.053 | 0.974 | 0.969 | 0.041 | 0.055 | -0.004 | 0.001 |
| England | 0.974 | 0.965 | 0.046 | 0.051 | 0.970 | 0.965 | 0.046 | 0.053 | -0.004 | 0.000 |
| Estonia | 0.974 | 0.965 | 0.045 | 0.050 | 0.973 | 0.968 | 0.043 | 0.051 | -0.001 | -0.002 |
| Finland | 0.985 | 0.980 | 0.044 | 0.043 | 0.982 | 0.979 | 0.046 | 0.044 | -0.003 | 0.002 |
| France | 0.975 | 0.966 | 0.036 | 0.040 | 0.974 | 0.969 | 0.034 | 0.041 | -0.001 | -0.002 |
| Georgia | 0.981 | 0.975 | 0.029 | 0.046 | 0.981 | 0.978 | 0.027 | 0.046 | 0.000 | -0.002 |
| Germany | 0.979 | 0.972 | 0.034 | 0.044 | 0.978 | 0.974 | 0.033 | 0.045 | -0.001 | -0.001 |
| Greece | 0.988 | 0.984 | 0.027 | 0.035 | 0.982 | 0.979 | 0.030 | 0.038 | -0.006 | 0.003 |
| Hungary | 0.968 | 0.957 | 0.039 | 0.050 | 0.962 | 0.955 | 0.040 | 0.053 | -0.006 | 0.001 |
| Iceland | 0.990 | 0.987 | 0.031 | 0.036 | 0.990 | 0.988 | 0.030 | 0.036 | 0.000 | -0.001 |
| Ireland | 0.967 | 0.956 | 0.055 | 0.053 | 0.963 | 0.956 | 0.055 | 0.054 | -0.004 | 0.000 |
| Israel | 0.972 | 0.963 | 0.034 | 0.040 | 0.970 | 0.965 | 0.034 | 0.042 | -0.002 | 0.000 |
| Italy | 0.979 | 0.971 | 0.036 | 0.042 | 0.980 | 0.976 | 0.033 | 0.043 | 0.001 | -0.003 |
| Kazakhstan | 0.983 | 0.977 | 0.034 | 0.043 | 0.983 | 0.980 | 0.032 | 0.044 | 0.000 | -0.002 |
| Latvia | 0.980 | 0.973 | 0.035 | 0.043 | 0.981 | 0.978 | 0.032 | 0.043 | 0.001 | -0.003 |
| Lithuania | 0.979 | 0.971 | 0.044 | 0.047 | 0.979 | 0.975 | 0.041 | 0.048 | 0.000 | -0.003 |
| Luxembourg | 0.977 | 0.970 | 0.036 | 0.044 | 0.977 | 0.973 | 0.034 | 0.045 | 0.000 | -0.002 |
| Malta | 0.985 | 0.980 | 0.031 | 0.042 | 0.983 | 0.980 | 0.030 | 0.043 | -0.002 | -0.001 |
| Netherlands | 0.990 | 0.986 | 0.021 | 0.037 | 0.991 | 0.989 | 0.018 | 0.038 | 0.001 | -0.003 |
| North Macedonia | 0.979 | 0.972 | 0.036 | 0.041 | 0.977 | 0.973 | 0.036 | 0.043 | -0.002 | 0.000 |
| Norway | 0.986 | 0.982 | 0.037 | 0.035 | 0.985 | 0.982 | 0.037 | 0.035 | -0.001 | 0.000 |
| Poland | 0.975 | 0.967 | 0.041 | 0.046 | 0.977 | 0.973 | 0.038 | 0.046 | 0.002 | -0.003 |
| Portugal | 0.976 | 0.967 | 0.046 | 0.045 | 0.973 | 0.969 | 0.045 | 0.045 | -0.003 | -0.001 |
| Republic of Moldova | 0.981 | 0.975 | 0.029 | 0.038 | 0.981 | 0.978 | 0.027 | 0.039 | 0.000 | -0.002 |
| Romania | 0.982 | 0.976 | 0.033 | 0.037 | 0.982 | 0.978 | 0.032 | 0.038 | 0.000 | -0.001 |
| Russian Federation | 0.989 | 0.986 | 0.035 | 0.033 | 0.988 | 0.986 | 0.034 | 0.034 | -0.001 | -0.001 |
| Scotland | 0.971 | 0.961 | 0.048 | 0.055 | 0.970 | 0.965 | 0.046 | 0.056 | -0.001 | -0.002 |
| Serbia | 0.975 | 0.967 | 0.049 | 0.053 | 0.975 | 0.971 | 0.046 | 0.054 | 0.000 | -0.003 |
| Slovenia | 0.985 | 0.980 | 0.034 | 0.038 | 0.986 | 0.983 | 0.031 | 0.039 | 0.001 | -0.003 |
| Spain | 0.982 | 0.976 | 0.047 | 0.042 | 0.982 | 0.978 | 0.044 | 0.042 | 0.000 | -0.003 |
| Sweden | 0.967 | 0.956 | 0.047 | 0.056 | 0.965 | 0.958 | 0.045 | 0.057 | -0.002 | -0.002 |
| Switzerland | 0.981 | 0.975 | 0.029 | 0.037 | 0.978 | 0.973 | 0.030 | 0.040 | -0.003 | 0.001 |
| Turkey | 0.981 | 0.975 | 0.038 | 0.038 | 0.980 | 0.977 | 0.036 | 0.039 | -0.001 | -0.002 |
| Ukraine | 0.973 | 0.964 | 0.043 | 0.047 | 0.974 | 0.969 | 0.040 | 0.047 | 0.001 | -0.003 |
| Wales | 0.979 | 0.973 | 0.047 | 0.040 | 0.977 | 0.973 | 0.046 | 0.041 | -0.002 | -0.001 |
| Notes: SMU = social media use; par. = number of free parameters; CFI = comparative fit index; TLI = Tucker Lewis index; RMSEA = root mean square error of approximation; SRMR = standardized root mean square residual.  ^1^ Item thresholds and factor loadings were allowed to vary across boys and girls.  ^2^ Item thresholds and factor loadings were constrained to be equal across boys and girls. | | | | | | | | | | |

| **Table S8: Age measurement invariance, by country (n = 221,093 in 44 countries)** | | | | | | | | | | |
| --- | --- | --- | --- | --- | --- | --- | --- | --- | --- | --- |
|  | **Configural invariance^1^ (par. = 54)** | | | | **Scalar invariance^2^ (par. = 40)** | | | | **Change** | |
|  | **CFI** | **TLI** | **RMSEA** | **SRMR** | **CFI** | **TLI** | **RMSEA** | **SRMR** | **ΔCFI** | **ΔRMSEA** |
| Albania | 0.988 | 0.985 | 0.027 | 0.054 | 0.982 | 0.979 | 0.031 | 0.058 | -0.006 | 0.004 |
| Armenia | 0.989 | 0.986 | 0.027 | 0.039 | 0.989 | 0.988 | 0.025 | 0.041 | 0.000 | -0.002 |
| Austria | 0.983 | 0.978 | 0.029 | 0.043 | 0.983 | 0.981 | 0.027 | 0.045 | 0.000 | -0.002 |
| Azerbaijan | 0.982 | 0.976 | 0.059 | 0.080 | 0.980 | 0.978 | 0.057 | 0.080 | -0.002 | -0.002 |
| Belgium (Flanders) | 0.978 | 0.971 | 0.038 | 0.047 | 0.978 | 0.975 | 0.035 | 0.048 | 0.000 | -0.003 |
| Belgium (Wallonia) | 0.980 | 0.973 | 0.038 | 0.042 | 0.976 | 0.973 | 0.038 | 0.045 | -0.004 | 0.000 |
| Canada | 0.967 | 0.956 | 0.032 | 0.052 | 0.968 | 0.963 | 0.030 | 0.053 | 0.001 | -0.002 |
| Croatia | 0.978 | 0.970 | 0.047 | 0.046 | 0.976 | 0.972 | 0.045 | 0.047 | -0.002 | -0.002 |
| Czechia | 0.978 | 0.971 | 0.037 | 0.044 | 0.978 | 0.975 | 0.035 | 0.045 | 0.000 | -0.002 |
| Denmark | 0.975 | 0.966 | 0.042 | 0.060 | 0.976 | 0.973 | 0.038 | 0.062 | 0.001 | -0.004 |
| England | 0.976 | 0.968 | 0.043 | 0.052 | 0.976 | 0.973 | 0.039 | 0.054 | 0.000 | -0.004 |
| Estonia | 0.973 | 0.964 | 0.045 | 0.054 | 0.969 | 0.965 | 0.044 | 0.057 | -0.004 | -0.001 |
| Finland | 0.988 | 0.984 | 0.039 | 0.046 | 0.988 | 0.986 | 0.037 | 0.046 | 0.000 | -0.002 |
| France | 0.978 | 0.971 | 0.033 | 0.041 | 0.977 | 0.974 | 0.032 | 0.043 | -0.001 | -0.001 |
| Georgia | 0.984 | 0.979 | 0.023 | 0.044 | 0.984 | 0.982 | 0.022 | 0.045 | 0.000 | -0.001 |
| Germany | 0.983 | 0.977 | 0.031 | 0.043 | 0.982 | 0.980 | 0.029 | 0.045 | -0.001 | -0.002 |
| Greece | 0.985 | 0.980 | 0.027 | 0.041 | 0.980 | 0.977 | 0.029 | 0.045 | -0.005 | 0.002 |
| Hungary | 0.963 | 0.951 | 0.041 | 0.057 | 0.959 | 0.953 | 0.040 | 0.061 | -0.004 | -0.001 |
| Iceland | 0.989 | 0.986 | 0.033 | 0.039 | 0.989 | 0.988 | 0.030 | 0.039 | 0.000 | -0.003 |
| Ireland | 0.966 | 0.955 | 0.054 | 0.055 | 0.964 | 0.959 | 0.052 | 0.057 | -0.002 | -0.002 |
| Israel | 0.974 | 0.965 | 0.033 | 0.041 | 0.976 | 0.972 | 0.029 | 0.042 | 0.002 | -0.004 |
| Italy | 0.978 | 0.971 | 0.035 | 0.046 | 0.976 | 0.973 | 0.034 | 0.047 | -0.002 | -0.001 |
| Kazakhstan | 0.982 | 0.976 | 0.032 | 0.047 | 0.982 | 0.980 | 0.029 | 0.049 | 0.000 | -0.003 |
| Latvia | 0.981 | 0.974 | 0.034 | 0.045 | 0.979 | 0.976 | 0.033 | 0.048 | -0.002 | -0.001 |
| Lithuania | 0.978 | 0.971 | 0.045 | 0.051 | 0.979 | 0.976 | 0.041 | 0.052 | 0.001 | -0.004 |
| Luxembourg | 0.978 | 0.970 | 0.036 | 0.045 | 0.977 | 0.974 | 0.033 | 0.047 | -0.001 | -0.003 |
| Malta | 0.985 | 0.980 | 0.029 | 0.043 | 0.972 | 0.968 | 0.037 | 0.048 | -0.013 | 0.008 |
| Netherlands | 0.989 | 0.986 | 0.021 | 0.044 | 0.990 | 0.989 | 0.018 | 0.048 | 0.001 | -0.003 |
| North Macedonia | 0.976 | 0.968 | 0.035 | 0.045 | 0.975 | 0.972 | 0.033 | 0.047 | -0.001 | -0.002 |
| Norway | 0.980 | 0.973 | 0.038 | 0.038 | 0.979 | 0.976 | 0.037 | 0.039 | -0.001 | -0.001 |
| Poland | 0.977 | 0.969 | 0.040 | 0.048 | 0.976 | 0.972 | 0.037 | 0.049 | -0.001 | -0.003 |
| Portugal | 0.977 | 0.970 | 0.043 | 0.046 | 0.975 | 0.971 | 0.043 | 0.048 | -0.002 | 0.000 |
| Republic of Moldova | 0.978 | 0.971 | 0.029 | 0.043 | 0.977 | 0.974 | 0.028 | 0.045 | -0.001 | -0.001 |
| Romania | 0.986 | 0.981 | 0.029 | 0.038 | 0.979 | 0.976 | 0.033 | 0.041 | -0.007 | 0.004 |
| Russian Federation | 0.989 | 0.986 | 0.033 | 0.035 | 0.987 | 0.985 | 0.034 | 0.037 | -0.002 | 0.001 |
| Scotland | 0.970 | 0.960 | 0.046 | 0.059 | 0.971 | 0.967 | 0.041 | 0.059 | 0.001 | -0.005 |
| Serbia | 0.979 | 0.971 | 0.043 | 0.052 | 0.979 | 0.976 | 0.039 | 0.053 | 0.000 | -0.004 |
| Slovenia | 0.985 | 0.981 | 0.032 | 0.042 | 0.985 | 0.983 | 0.030 | 0.045 | 0.000 | -0.002 |
| Spain | 0.983 | 0.977 | 0.045 | 0.042 | 0.981 | 0.978 | 0.044 | 0.044 | -0.002 | -0.001 |
| Sweden | 0.971 | 0.962 | 0.044 | 0.056 | 0.973 | 0.969 | 0.039 | 0.058 | 0.002 | -0.005 |
| Switzerland | 0.979 | 0.972 | 0.031 | 0.041 | 0.978 | 0.975 | 0.029 | 0.043 | -0.001 | -0.002 |
| Turkey | 0.978 | 0.971 | 0.040 | 0.044 | 0.977 | 0.974 | 0.038 | 0.045 | -0.001 | -0.002 |
| Ukraine | 0.976 | 0.968 | 0.040 | 0.048 | 0.974 | 0.970 | 0.039 | 0.050 | -0.002 | -0.001 |
| Wales | 0.981 | 0.975 | 0.046 | 0.041 | 0.980 | 0.978 | 0.043 | 0.041 | -0.001 | -0.003 |
| Notes: SMU = social media use; par. = number of free parameters; CFI = comparative fit index; TLI = Tucker Lewis index; RMSEA = root mean square error of approximation; SRMR = standardized root mean square residual. The sample size for this analysis (*n* = 221,093) differs from the other analyses (*n* = 222,532) and corresponds to the number of cases with complete data on age: multiple CFA can only be conducted on complete data on the grouping variable (age).  ^1^ Item thresholds and factor loadings were allowed to vary across 11-, 13-, and 15-year-olds.  ^2^ Item thresholds and factor loadings were constrained to be equal across 11-, 13-, and 15-year-olds. | | | | | | | | | | |

| **Table S9: Socioeconomic measurement invariance, by country (n = 212,353 in 44 countries)** | | | | | | | | | | |
| --- | --- | --- | --- | --- | --- | --- | --- | --- | --- | --- |
|  | **Configural invariance^1^ (par. = 54)** | | | | **Scalar invariance^2^ (par. = 40)** | | | | **Change** | |
|  | **CFI** | **TLI** | **RMSEA** | **SRMR** | **CFI** | **TLI** | **RMSEA** | **SRMR** | **ΔCFI** | **ΔRMSEA** |
| Albania | 0.992 | 0.989 | 0.025 | 0.047 | 0.991 | 0.990 | 0.023 | 0.049 | -0.001 | -0.002 |
| Armenia | 0.988 | 0.984 | 0.030 | 0.040 | 0.989 | 0.988 | 0.026 | 0.040 | 0.001 | -0.004 |
| Austria | 0.984 | 0.978 | 0.030 | 0.045 | 0.985 | 0.983 | 0.026 | 0.047 | 0.001 | -0.004 |
| Azerbaijan | 0.977 | 0.969 | 0.049 | 0.060 | 0.977 | 0.974 | 0.045 | 0.062 | 0.000 | -0.004 |
| Belgium (Flanders) | 0.980 | 0.973 | 0.037 | 0.045 | 0.982 | 0.980 | 0.032 | 0.046 | 0.002 | -0.005 |
| Belgium (Wallonia) | 0.982 | 0.976 | 0.037 | 0.041 | 0.983 | 0.980 | 0.033 | 0.043 | 0.001 | -0.004 |
| Canada | 0.974 | 0.965 | 0.034 | 0.052 | 0.973 | 0.970 | 0.032 | 0.054 | -0.001 | -0.002 |
| Croatia | 0.978 | 0.970 | 0.047 | 0.047 | 0.978 | 0.975 | 0.043 | 0.047 | 0.000 | -0.004 |
| Czechia | 0.977 | 0.969 | 0.038 | 0.044 | 0.978 | 0.975 | 0.034 | 0.045 | 0.001 | -0.004 |
| Denmark | 0.973 | 0.964 | 0.044 | 0.063 | 0.977 | 0.973 | 0.038 | 0.063 | 0.004 | -0.006 |
| England | 0.975 | 0.967 | 0.045 | 0.054 | 0.976 | 0.972 | 0.041 | 0.055 | 0.001 | -0.004 |
| Estonia | 0.978 | 0.971 | 0.041 | 0.050 | 0.981 | 0.978 | 0.036 | 0.051 | 0.003 | -0.005 |
| Finland | 0.987 | 0.983 | 0.040 | 0.043 | 0.987 | 0.986 | 0.037 | 0.044 | 0.000 | -0.003 |
| France | 0.977 | 0.969 | 0.036 | 0.044 | 0.979 | 0.976 | 0.032 | 0.045 | 0.002 | -0.004 |
| Georgia | 0.986 | 0.981 | 0.025 | 0.045 | 0.987 | 0.985 | 0.022 | 0.046 | 0.001 | -0.003 |
| Germany | 0.982 | 0.976 | 0.032 | 0.044 | 0.984 | 0.982 | 0.028 | 0.045 | 0.002 | -0.004 |
| Greece | 0.985 | 0.980 | 0.029 | 0.040 | 0.985 | 0.983 | 0.027 | 0.042 | 0.000 | -0.002 |
| Hungary | 0.962 | 0.949 | 0.041 | 0.057 | 0.967 | 0.962 | 0.036 | 0.059 | 0.005 | -0.005 |
| Iceland | 0.993 | 0.990 | 0.028 | 0.036 | 0.993 | 0.992 | 0.026 | 0.036 | 0.000 | -0.002 |
| Ireland | 0.966 | 0.955 | 0.056 | 0.057 | 0.966 | 0.961 | 0.052 | 0.057 | 0.000 | -0.004 |
| Israel | 0.973 | 0.964 | 0.035 | 0.041 | 0.973 | 0.970 | 0.032 | 0.043 | 0.000 | -0.003 |
| Italy | 0.979 | 0.973 | 0.036 | 0.045 | 0.982 | 0.980 | 0.031 | 0.045 | 0.003 | -0.005 |
| Kazakhstan | 0.981 | 0.975 | 0.034 | 0.048 | 0.980 | 0.977 | 0.033 | 0.050 | -0.001 | -0.001 |
| Latvia | 0.983 | 0.977 | 0.033 | 0.043 | 0.985 | 0.983 | 0.028 | 0.044 | 0.002 | -0.005 |
| Lithuania | 0.980 | 0.973 | 0.043 | 0.049 | 0.982 | 0.979 | 0.038 | 0.050 | 0.002 | -0.005 |
| Luxembourg | 0.976 | 0.967 | 0.037 | 0.048 | 0.975 | 0.971 | 0.035 | 0.050 | -0.001 | -0.002 |
| Malta | 0.989 | 0.985 | 0.027 | 0.044 | 0.989 | 0.988 | 0.024 | 0.045 | 0.000 | -0.003 |
| Netherlands | 0.987 | 0.983 | 0.023 | 0.044 | 0.989 | 0.988 | 0.020 | 0.045 | 0.002 | -0.003 |
| North Macedonia | 0.976 | 0.967 | 0.038 | 0.046 | 0.976 | 0.973 | 0.035 | 0.047 | 0.000 | -0.003 |
| Norway | 0.987 | 0.982 | 0.037 | 0.037 | 0.987 | 0.985 | 0.034 | 0.038 | 0.000 | -0.003 |
| Poland | 0.978 | 0.971 | 0.041 | 0.048 | 0.980 | 0.977 | 0.036 | 0.049 | 0.002 | -0.005 |
| Portugal | 0.977 | 0.970 | 0.044 | 0.045 | 0.979 | 0.976 | 0.039 | 0.046 | 0.002 | -0.005 |
| Republic of Moldova | 0.986 | 0.981 | 0.025 | 0.037 | 0.987 | 0.985 | 0.023 | 0.039 | 0.001 | -0.002 |
| Romania | 0.984 | 0.978 | 0.032 | 0.040 | 0.985 | 0.983 | 0.028 | 0.040 | 0.001 | -0.004 |
| Russian Federation | 0.990 | 0.987 | 0.033 | 0.036 | 0.991 | 0.990 | 0.029 | 0.036 | 0.001 | -0.004 |
| Scotland | 0.972 | 0.963 | 0.047 | 0.057 | 0.972 | 0.968 | 0.043 | 0.058 | 0.000 | -0.004 |
| Serbia | 0.977 | 0.970 | 0.046 | 0.053 | 0.979 | 0.976 | 0.041 | 0.053 | 0.002 | -0.005 |
| Slovenia | 0.990 | 0.986 | 0.029 | 0.037 | 0.991 | 0.990 | 0.025 | 0.038 | 0.001 | -0.004 |
| Spain | 0.984 | 0.979 | 0.045 | 0.042 | 0.984 | 0.982 | 0.041 | 0.042 | 0.000 | -0.004 |
| Sweden | 0.971 | 0.961 | 0.046 | 0.058 | 0.969 | 0.965 | 0.044 | 0.061 | -0.002 | -0.002 |
| Switzerland | 0.978 | 0.970 | 0.032 | 0.042 | 0.979 | 0.976 | 0.029 | 0.044 | 0.001 | -0.003 |
| Turkey | 0.985 | 0.980 | 0.035 | 0.039 | 0.986 | 0.984 | 0.031 | 0.039 | 0.001 | -0.004 |
| Ukraine | 0.976 | 0.968 | 0.040 | 0.046 | 0.977 | 0.974 | 0.036 | 0.047 | 0.001 | -0.004 |
| Wales | 0.981 | 0.975 | 0.048 | 0.040 | 0.981 | 0.978 | 0.044 | 0.041 | 0.000 | -0.004 |
| Notes: SMU = social media use; par. = number of free parameters; CFI = comparative fit index; TLI = Tucker Lewis index; RMSEA = root mean square error of approximation; SRMR = standardized root mean square residual. The sample size for this analysis (*n* = 212,353) differs from the other analyses (*n* = 222,532) and corresponds to the number of cases with complete data on socioeconomic status (i.e., family affluence): multiple CFA can only be conducted on complete data on the grouping variable (socioeconomic status).  ^1^ Item thresholds and factor loadings were allowed to vary across adolescents with low, moderate, and high socioeconomic status.  ^2^ Item thresholds and factor loadings were constrained to be equal across adolescents with low, moderate, and high socioeconomic status. | | | | | | | | | | |

| **Table S10: Life satisfaction, by problematic SMU and country (n = 222,532 in 44 countries)** | | | | | | | | | | | | |
| --- | --- | --- | --- | --- | --- | --- | --- | --- | --- | --- | --- | --- |
|  | **Means** | | | | | | | | | **Effect size mean differences** | | |
|  | **Total** | | | **Non-problematic** | | | **Problematic** | | |  |  |  |
|  | **Mean** | **95% LL** | **95% UL** | **Mean** | **95% LL** | **95% UL** | **Mean** | **95% LL** | **95% UL** | ***β*** | ***SE*** | ***p*** |
| Albania | 8.15 | 8.05 | 8.25 | 8.22 | 8.12 | 8.33 | 7.61 | 7.29 | 7.93 | -0.193 | 0.084 | 0.021 |
| Armenia | 8.34 | 8.29 | 8.39 | 8.37 | 8.31 | 8.42 | 7.94 | 7.66 | 8.22 | -0.242 | 0.073 | 0.001 |
| Austria | 7.70 | 7.64 | 7.77 | 7.76 | 7.70 | 7.82 | 6.58 | 6.20 | 6.96 | -0.517 | 0.092 | <0.001 |
| Azerbaijan | 8.34 | 8.27 | 8.40 | 8.34 | 8.28 | 8.41 | 8.30 | 8.07 | 8.52 | -0.118 | 0.076 | 0.121 |
| Belgium (Flanders) | 7.79 | 7.75 | 7.84 | 7.84 | 7.80 | 7.88 | 7.15 | 6.94 | 7.36 | -0.428 | 0.072 | <0.001 |
| Belgium (Wallonia) | 7.55 | 7.50 | 7.60 | 7.62 | 7.57 | 7.68 | 6.73 | 6.51 | 6.94 | -0.455 | 0.061 | <0.001 |
| Canada | 7.30 | 7.25 | 7.35 | 7.38 | 7.32 | 7.43 | 6.13 | 5.84 | 6.42 | -0.551 | 0.071 | <0.001 |
| Croatia | 8.09 | 8.04 | 8.14 | 8.18 | 8.13 | 8.24 | 7.24 | 7.03 | 7.44 | -0.461 | 0.058 | <0.001 |
| Czechia | 7.79 | 7.75 | 7.82 | 7.83 | 7.80 | 7.86 | 7.00 | 6.81 | 7.19 | -0.423 | 0.057 | <0.001 |
| Denmark | 7.68 | 7.62 | 7.74 | 7.72 | 7.66 | 7.78 | 6.71 | 6.34 | 7.07 | -0.536 | 0.104 | <0.001 |
| England | 7.44 | 7.37 | 7.51 | 7.55 | 7.48 | 7.62 | 6.14 | 5.82 | 6.46 | -0.682 | 0.092 | <0.001 |
| Estonia | 7.72 | 7.66 | 7.77 | 7.78 | 7.73 | 7.83 | 6.73 | 6.43 | 7.02 | -0.550 | 0.085 | <0.001 |
| Finland | 7.83 | 7.77 | 7.90 | 7.93 | 7.86 | 7.99 | 7.01 | 6.73 | 7.30 | -0.466 | 0.088 | <0.001 |
| France | 7.65 | 7.61 | 7.70 | 7.72 | 7.67 | 7.76 | 6.89 | 6.70 | 7.08 | -0.433 | 0.058 | <0.001 |
| Georgia | 7.99 | 7.93 | 8.05 | 8.00 | 7.93 | 8.06 | 7.81 | 7.49 | 8.13 | -0.089 | 0.101 | 0.374 |
| Germany | 7.68 | 7.63 | 7.73 | 7.71 | 7.65 | 7.76 | 7.14 | 6.78 | 7.49 | -0.324 | 0.099 | 0.001 |
| Greece | 7.52 | 7.46 | 7.58 | 7.63 | 7.57 | 7.70 | 6.49 | 6.26 | 6.71 | -0.475 | 0.058 | <0.001 |
| Hungary | 7.59 | 7.52 | 7.65 | 7.65 | 7.58 | 7.71 | 6.53 | 6.19 | 6.86 | -0.560 | 0.086 | <0.001 |
| Iceland | 7.61 | 7.56 | 7.65 | 7.66 | 7.61 | 7.70 | 6.57 | 6.27 | 6.87 | -0.515 | 0.074 | <0.001 |
| Ireland | 7.52 | 7.46 | 7.58 | 7.63 | 7.56 | 7.69 | 6.76 | 6.57 | 6.95 | -0.371 | 0.058 | <0.001 |
| Israel | 7.81 | 7.75 | 7.87 | 7.87 | 7.81 | 7.93 | 6.64 | 6.11 | 7.18 | -0.478 | 0.113 | <0.001 |
| Italy | 7.58 | 7.52 | 7.64 | 7.68 | 7.62 | 7.73 | 6.79 | 6.59 | 6.99 | -0.480 | 0.056 | <0.001 |
| Kazakhstan | 8.55 | 8.50 | 8.60 | 8.56 | 8.51 | 8.62 | 8.28 | 7.99 | 8.58 | -0.141 | 0.080 | 0.079 |
| Latvia | 7.39 | 7.33 | 7.45 | 7.45 | 7.39 | 7.51 | 6.31 | 6.00 | 6.62 | -0.529 | 0.087 | <0.001 |
| Lithuania | 7.91 | 7.85 | 7.97 | 7.97 | 7.91 | 8.04 | 7.14 | 6.86 | 7.41 | -0.437 | 0.068 | <0.001 |
| Luxembourg | 7.63 | 7.58 | 7.69 | 7.68 | 7.63 | 7.74 | 7.01 | 6.72 | 7.29 | -0.342 | 0.079 | <0.001 |
| Malta | 7.30 | 7.22 | 7.38 | 7.40 | 7.31 | 7.49 | 6.79 | 6.58 | 7.00 | -0.247 | 0.061 | <0.001 |
| Netherlands | 7.77 | 7.73 | 7.82 | 7.81 | 7.76 | 7.86 | 6.71 | 6.34 | 7.08 | -0.627 | 0.110 | <0.001 |
| North Macedonia | 8.42 | 8.36 | 8.48 | 8.51 | 8.45 | 8.57 | 7.59 | 7.36 | 7.82 | -0.368 | 0.061 | <0.001 |
| Norway | 7.89 | 7.83 | 7.96 | 7.94 | 7.88 | 8.01 | 7.41 | 7.14 | 7.68 | -0.257 | 0.078 | 0.001 |
| Poland | 7.48 | 7.43 | 7.54 | 7.56 | 7.51 | 7.62 | 6.55 | 6.32 | 6.78 | -0.458 | 0.061 | <0.001 |
| Portugal | 7.74 | 7.70 | 7.79 | 7.78 | 7.73 | 7.83 | 7.16 | 6.91 | 7.41 | -0.315 | 0.071 | <0.001 |
| Republic of Moldova | 8.25 | 8.20 | 8.30 | 8.26 | 8.21 | 8.31 | 8.12 | 7.93 | 8.31 | -0.037 | 0.063 | 0.554 |
| Romania | 8.32 | 8.28 | 8.37 | 8.39 | 8.34 | 8.44 | 7.88 | 7.73 | 8.03 | -0.266 | 0.048 | <0.001 |
| Russian Federation | 7.40 | 7.34 | 7.47 | 7.44 | 7.38 | 7.51 | 6.91 | 6.63 | 7.19 | -0.267 | 0.064 | <0.001 |
| Scotland | 7.63 | 7.57 | 7.69 | 7.72 | 7.66 | 7.78 | 6.79 | 6.55 | 7.02 | -0.415 | 0.063 | <0.001 |
| Serbia | 8.26 | 8.20 | 8.32 | 8.32 | 8.26 | 8.38 | 7.47 | 7.20 | 7.74 | -0.402 | 0.079 | <0.001 |
| Slovenia | 7.95 | 7.90 | 8.00 | 8.00 | 7.95 | 8.05 | 7.04 | 6.78 | 7.29 | -0.454 | 0.078 | <0.001 |
| Spain | 8.06 | 8.01 | 8.12 | 8.14 | 8.08 | 8.19 | 7.62 | 7.44 | 7.79 | -0.266 | 0.051 | <0.001 |
| Sweden | 7.45 | 7.39 | 7.51 | 7.52 | 7.45 | 7.58 | 6.20 | 5.86 | 6.54 | -0.590 | 0.083 | <0.001 |
| Switzerland | 7.67 | 7.62 | 7.71 | 7.72 | 7.68 | 7.77 | 6.43 | 6.15 | 6.70 | -0.680 | 0.073 | <0.001 |
| Turkey | 6.61 | 6.55 | 6.68 | 6.67 | 6.61 | 6.74 | 6.13 | 5.94 | 6.32 | -0.210 | 0.042 | <0.001 |
| Ukraine | 7.71 | 7.66 | 7.75 | 7.75 | 7.70 | 7.80 | 7.11 | 6.89 | 7.33 | -0.332 | 0.061 | <0.001 |
| Wales | 7.59 | 7.56 | 7.62 | 7.71 | 7.68 | 7.75 | 6.69 | 6.57 | 6.81 | -0.462 | 0.033 | <0.001 |
| Notes: SMU = social media use; LL = confidence interval lower limit; UL = confidence interval upper limit; *β* = STDY-standardized (i.e., *B*/standard deviation(Y)), controlled for gender, age, and socioeconomic status; *SE* = standard error; *p* = p-value. | | | | | | | | | | | | |

| **Table S11: Psychosomatic complaints, by problematic SMU and country (n = 222,532 in 44 countries)** | | | | | | | | | | | | |
| --- | --- | --- | --- | --- | --- | --- | --- | --- | --- | --- | --- | --- |
|  | **Means** | | | | | | | | | **Effect size mean differences** | | |
|  | **Total** | | | **Non-problematic** | | | **Problematic** | | |  |  |  |
|  | **Mean** | **95% LL** | **95% UL** | **Mean** | **95% LL** | **95% UL** | **Mean** | **95% LL** | **95% UL** | ***β*** | ***SE*** | ***p*** |
| Albania | 1.89 | 1.85 | 1.93 | 1.82 | 1.78 | 1.86 | 2.40 | 2.27 | 2.53 | 0.623 | 0.088 | <0.001 |
| Armenia | 1.86 | 1.84 | 1.88 | 1.83 | 1.81 | 1.85 | 2.31 | 2.19 | 2.42 | 0.634 | 0.082 | <0.001 |
| Austria | 1.99 | 1.97 | 2.02 | 1.96 | 1.94 | 1.99 | 2.65 | 2.50 | 2.79 | 0.806 | 0.081 | <0.001 |
| Azerbaijan | 1.67 | 1.65 | 1.70 | 1.60 | 1.57 | 1.63 | 2.35 | 2.26 | 2.44 | 0.924 | 0.074 | <0.001 |
| Belgium (Flanders) | 1.99 | 1.97 | 2.01 | 1.95 | 1.93 | 1.97 | 2.50 | 2.39 | 2.61 | 0.714 | 0.077 | <0.001 |
| Belgium (Wallonia) | 2.22 | 2.20 | 2.24 | 2.18 | 2.15 | 2.20 | 2.68 | 2.60 | 2.76 | 0.574 | 0.049 | <0.001 |
| Canada | 2.09 | 2.07 | 2.12 | 2.05 | 2.03 | 2.07 | 2.74 | 2.63 | 2.86 | 0.699 | 0.069 | <0.001 |
| Croatia | 1.89 | 1.87 | 1.92 | 1.83 | 1.80 | 1.85 | 2.46 | 2.38 | 2.55 | 0.714 | 0.055 | <0.001 |
| Czechia | 2.05 | 2.03 | 2.07 | 2.02 | 2.01 | 2.04 | 2.56 | 2.47 | 2.64 | 0.669 | 0.060 | <0.001 |
| Denmark | 1.97 | 1.95 | 2.00 | 1.95 | 1.92 | 1.98 | 2.52 | 2.34 | 2.69 | 0.688 | 0.112 | <0.001 |
| England | 2.18 | 2.15 | 2.21 | 2.13 | 2.09 | 2.16 | 2.77 | 2.63 | 2.91 | 0.666 | 0.089 | <0.001 |
| Estonia | 2.16 | 2.13 | 2.19 | 2.12 | 2.09 | 2.14 | 2.86 | 2.74 | 2.98 | 0.795 | 0.064 | <0.001 |
| Finland | 2.22 | 2.19 | 2.25 | 2.16 | 2.13 | 2.20 | 2.70 | 2.59 | 2.82 | 0.598 | 0.068 | <0.001 |
| France | 2.20 | 2.18 | 2.22 | 2.16 | 2.14 | 2.18 | 2.74 | 2.66 | 2.82 | 0.671 | 0.055 | <0.001 |
| Georgia | 2.06 | 2.03 | 2.09 | 2.04 | 2.02 | 2.07 | 2.42 | 2.27 | 2.58 | 0.408 | 0.090 | <0.001 |
| Germany | 2.03 | 2.01 | 2.05 | 2.00 | 1.98 | 2.03 | 2.52 | 2.39 | 2.65 | 0.713 | 0.094 | <0.001 |
| Greece | 2.16 | 2.13 | 2.18 | 2.09 | 2.06 | 2.12 | 2.78 | 2.69 | 2.87 | 0.713 | 0.058 | <0.001 |
| Hungary | 2.21 | 2.18 | 2.24 | 2.17 | 2.14 | 2.20 | 2.90 | 2.77 | 3.04 | 0.816 | 0.088 | <0.001 |
| Iceland | 2.20 | 2.18 | 2.22 | 2.17 | 2.15 | 2.20 | 2.69 | 2.57 | 2.82 | 0.564 | 0.065 | <0.001 |
| Ireland | 2.04 | 2.01 | 2.07 | 1.97 | 1.94 | 2.00 | 2.56 | 2.48 | 2.65 | 0.638 | 0.056 | <0.001 |
| Israel | 2.40 | 2.38 | 2.43 | 2.36 | 2.33 | 2.38 | 3.26 | 3.12 | 3.41 | 0.872 | 0.072 | <0.001 |
| Italy | 2.42 | 2.39 | 2.45 | 2.36 | 2.34 | 2.39 | 2.89 | 2.81 | 2.98 | 0.573 | 0.054 | <0.001 |
| Kazakhstan | 1.72 | 1.70 | 1.75 | 1.71 | 1.68 | 1.73 | 2.06 | 1.92 | 2.20 | 0.453 | 0.094 | <0.001 |
| Latvia | 2.14 | 2.11 | 2.16 | 2.10 | 2.08 | 2.13 | 2.75 | 2.61 | 2.89 | 0.663 | 0.078 | <0.001 |
| Lithuania | 1.99 | 1.96 | 2.01 | 1.94 | 1.91 | 1.97 | 2.49 | 2.38 | 2.61 | 0.630 | 0.058 | <0.001 |
| Luxembourg | 2.21 | 2.18 | 2.23 | 2.17 | 2.14 | 2.19 | 2.68 | 2.58 | 2.78 | 0.635 | 0.068 | <0.001 |
| Malta | 2.37 | 2.33 | 2.41 | 2.27 | 2.23 | 2.30 | 2.90 | 2.80 | 3.00 | 0.645 | 0.062 | <0.001 |
| Netherlands | 1.91 | 1.89 | 1.94 | 1.89 | 1.87 | 1.91 | 2.61 | 2.44 | 2.78 | 0.859 | 0.111 | <0.001 |
| North Macedonia | 1.78 | 1.75 | 1.81 | 1.72 | 1.69 | 1.75 | 2.36 | 2.26 | 2.46 | 0.763 | 0.062 | <0.001 |
| Norway | 1.92 | 1.90 | 1.95 | 1.90 | 1.87 | 1.93 | 2.15 | 2.04 | 2.26 | 0.309 | 0.079 | <0.001 |
| Poland | 2.14 | 2.11 | 2.16 | 2.09 | 2.06 | 2.11 | 2.73 | 2.64 | 2.82 | 0.710 | 0.060 | <0.001 |
| Portugal | 1.91 | 1.89 | 1.93 | 1.88 | 1.85 | 1.90 | 2.44 | 2.33 | 2.55 | 0.656 | 0.066 | <0.001 |
| Republic of Moldova | 2.01 | 1.98 | 2.03 | 1.98 | 1.96 | 2.01 | 2.27 | 2.17 | 2.36 | 0.322 | 0.068 | <0.001 |
| Romania | 2.12 | 2.09 | 2.14 | 2.04 | 2.02 | 2.07 | 2.62 | 2.54 | 2.69 | 0.603 | 0.046 | <0.001 |
| Russian Federation | 1.98 | 1.95 | 2.00 | 1.94 | 1.91 | 1.97 | 2.43 | 2.31 | 2.55 | 0.559 | 0.068 | <0.001 |
| Scotland | 2.06 | 2.03 | 2.08 | 2.00 | 1.97 | 2.02 | 2.64 | 2.54 | 2.74 | 0.670 | 0.064 | <0.001 |
| Serbia | 1.86 | 1.84 | 1.89 | 1.82 | 1.80 | 1.85 | 2.40 | 2.27 | 2.52 | 0.635 | 0.093 | <0.001 |
| Slovenia | 1.91 | 1.89 | 1.94 | 1.88 | 1.86 | 1.90 | 2.55 | 2.43 | 2.66 | 0.731 | 0.069 | <0.001 |
| Spain | 1.81 | 1.79 | 1.83 | 1.76 | 1.74 | 1.79 | 2.10 | 2.03 | 2.18 | 0.446 | 0.046 | <0.001 |
| Sweden | 2.32 | 2.29 | 2.35 | 2.28 | 2.26 | 2.31 | 2.98 | 2.84 | 3.12 | 0.718 | 0.086 | <0.001 |
| Switzerland | 2.08 | 2.06 | 2.10 | 2.05 | 2.03 | 2.07 | 2.74 | 2.64 | 2.84 | 0.885 | 0.064 | <0.001 |
| Turkey | 2.44 | 2.42 | 2.47 | 2.39 | 2.36 | 2.41 | 2.90 | 2.83 | 2.97 | 0.527 | 0.043 | <0.001 |
| Ukraine | 2.16 | 2.14 | 2.18 | 2.12 | 2.10 | 2.14 | 2.62 | 2.53 | 2.71 | 0.606 | 0.062 | <0.001 |
| Wales | 2.10 | 2.09 | 2.12 | 2.02 | 2.01 | 2.04 | 2.68 | 2.63 | 2.73 | 0.692 | 0.030 | <0.001 |
| Notes: SMU = social media use; LL = confidence interval lower limit; UL = confidence interval upper limit; *β* = STDY-standardized (i.e., *B*/standard deviation(Y)), controlled for gender, age, and socioeconomic status; *SE* = standard error; *p* = p-value. | | | | | | | | | | | | |

| **Table S12: Intensity of online communication, by problematic SMU and country (n = 222,532 in 44 countries)** | | | | | | | | | | | | |
| --- | --- | --- | --- | --- | --- | --- | --- | --- | --- | --- | --- | --- |
|  | **Means** | | | | | | | | | **Effect size mean differences** | | |
|  | **Total** | | | **Non-problematic** | | | **Problematic** | | |  |  |  |
|  | **Mean** | **95% LL** | **95% UL** | **Mean** | **95% LL** | **95% UL** | **Mean** | **95% LL** | **95% UL** | ***β*** | ***SE*** | ***p*** |
| Albania | 4.04 | 3.98 | 4.10 | 4.01 | 3.94 | 4.07 | 4.31 | 4.14 | 4.47 | 0.201 | 0.079 | 0.011 |
| Armenia | 3.58 | 3.53 | 3.62 | 3.56 | 3.52 | 3.61 | 3.79 | 3.61 | 3.96 | 0.163 | 0.072 | 0.023 |
| Austria | 3.87 | 3.83 | 3.90 | 3.84 | 3.81 | 3.88 | 4.37 | 4.25 | 4.49 | 0.470 | 0.059 | <0.001 |
| Azerbaijan | 2.79 | 2.74 | 2.84 | 2.84 | 2.79 | 2.89 | 2.33 | 2.16 | 2.49 | -0.273 | 0.086 | 0.001 |
| Belgium (Flanders) | 3.71 | 3.67 | 3.76 | 3.68 | 3.64 | 3.72 | 4.20 | 4.06 | 4.33 | 0.323 | 0.058 | <0.001 |
| Belgium (Wallonia) | 3.91 | 3.87 | 3.94 | 3.86 | 3.82 | 3.89 | 4.41 | 4.32 | 4.51 | 0.416 | 0.042 | <0.001 |
| Canada | 3.71 | 3.68 | 3.74 | 3.68 | 3.64 | 3.71 | 4.17 | 4.06 | 4.29 | 0.341 | 0.054 | <0.001 |
| Croatia | 3.73 | 3.69 | 3.77 | 3.69 | 3.65 | 3.73 | 4.11 | 4.00 | 4.22 | 0.282 | 0.051 | <0.001 |
| Czechia | 3.40 | 3.38 | 3.42 | 3.36 | 3.34 | 3.39 | 4.09 | 3.99 | 4.18 | 0.538 | 0.045 | <0.001 |
| Denmark | 3.86 | 3.82 | 3.89 | 3.84 | 3.80 | 3.88 | 4.17 | 3.98 | 4.37 | 0.252 | 0.097 | 0.009 |
| England | 3.76 | 3.72 | 3.80 | 3.71 | 3.67 | 3.76 | 4.29 | 4.14 | 4.44 | 0.440 | 0.072 | <0.001 |
| Estonia | 3.81 | 3.78 | 3.84 | 3.79 | 3.76 | 3.82 | 4.16 | 4.03 | 4.29 | 0.343 | 0.060 | <0.001 |
| Finland | 3.83 | 3.79 | 3.87 | 3.79 | 3.75 | 3.83 | 4.16 | 4.03 | 4.29 | 0.317 | 0.067 | <0.001 |
| France | 3.84 | 3.81 | 3.87 | 3.80 | 3.77 | 3.83 | 4.30 | 4.21 | 4.40 | 0.367 | 0.046 | <0.001 |
| Georgia | 3.64 | 3.60 | 3.67 | 3.63 | 3.59 | 3.67 | 3.75 | 3.56 | 3.94 | 0.082 | 0.083 | 0.328 |
| Germany | 3.62 | 3.58 | 3.66 | 3.60 | 3.56 | 3.63 | 4.05 | 3.89 | 4.21 | 0.392 | 0.078 | <0.001 |
| Greece | 3.73 | 3.69 | 3.77 | 3.68 | 3.64 | 3.72 | 4.19 | 4.08 | 4.30 | 0.327 | 0.049 | <0.001 |
| Hungary | 3.75 | 3.72 | 3.79 | 3.73 | 3.70 | 3.76 | 4.22 | 4.09 | 4.36 | 0.506 | 0.071 | <0.001 |
| Iceland | 3.79 | 3.76 | 3.81 | 3.77 | 3.74 | 3.80 | 4.16 | 4.03 | 4.29 | 0.266 | 0.063 | <0.001 |
| Ireland | 3.85 | 3.81 | 3.89 | 3.79 | 3.75 | 3.83 | 4.31 | 4.22 | 4.41 | 0.354 | 0.046 | <0.001 |
| Israel | 3.85 | 3.82 | 3.89 | 3.84 | 3.80 | 3.87 | 4.15 | 4.01 | 4.29 | 0.228 | 0.063 | <0.001 |
| Italy | 4.15 | 4.11 | 4.18 | 4.12 | 4.08 | 4.15 | 4.36 | 4.26 | 4.45 | 0.206 | 0.044 | <0.001 |
| Kazakhstan | 3.53 | 3.48 | 3.57 | 3.51 | 3.46 | 3.55 | 3.92 | 3.70 | 4.14 | 0.284 | 0.078 | <0.001 |
| Latvia | 3.46 | 3.43 | 3.50 | 3.44 | 3.40 | 3.48 | 3.92 | 3.75 | 4.08 | 0.382 | 0.069 | <0.001 |
| Lithuania | 3.93 | 3.89 | 3.96 | 3.91 | 3.87 | 3.94 | 4.16 | 4.03 | 4.30 | 0.212 | 0.058 | <0.001 |
| Luxembourg | 3.78 | 3.75 | 3.82 | 3.75 | 3.71 | 3.79 | 4.25 | 4.13 | 4.36 | 0.397 | 0.053 | <0.001 |
| Malta | 4.02 | 3.97 | 4.06 | 3.97 | 3.92 | 4.02 | 4.27 | 4.17 | 4.38 | 0.254 | 0.060 | <0.001 |
| Netherlands | 3.71 | 3.68 | 3.74 | 3.69 | 3.66 | 3.73 | 4.18 | 4.00 | 4.36 | 0.339 | 0.079 | <0.001 |
| North Macedonia | 4.10 | 4.07 | 4.13 | 4.07 | 4.04 | 4.11 | 4.37 | 4.27 | 4.46 | 0.220 | 0.052 | <0.001 |
| Norway | 3.86 | 3.81 | 3.90 | 3.84 | 3.79 | 3.88 | 4.06 | 3.91 | 4.20 | 0.166 | 0.060 | 0.005 |
| Poland | 3.98 | 3.95 | 4.01 | 3.96 | 3.92 | 3.99 | 4.31 | 4.21 | 4.41 | 0.264 | 0.047 | <0.001 |
| Portugal | 3.93 | 3.90 | 3.96 | 3.91 | 3.88 | 3.94 | 4.28 | 4.16 | 4.41 | 0.280 | 0.054 | <0.001 |
| Republic of Moldova | 3.85 | 3.81 | 3.88 | 3.81 | 3.77 | 3.85 | 4.28 | 4.16 | 4.40 | 0.343 | 0.049 | <0.001 |
| Romania | 4.05 | 4.02 | 4.09 | 4.01 | 3.98 | 4.05 | 4.31 | 4.22 | 4.40 | 0.234 | 0.046 | <0.001 |
| Russian Federation | 3.78 | 3.74 | 3.82 | 3.77 | 3.73 | 3.81 | 3.92 | 3.79 | 4.05 | 0.115 | 0.059 | 0.051 |
| Scotland | 3.94 | 3.90 | 3.97 | 3.89 | 3.85 | 3.92 | 4.45 | 4.35 | 4.54 | 0.469 | 0.046 | <0.001 |
| Serbia | 4.00 | 3.96 | 4.04 | 3.97 | 3.93 | 4.01 | 4.44 | 4.30 | 4.58 | 0.301 | 0.065 | <0.001 |
| Slovenia | 3.58 | 3.55 | 3.62 | 3.55 | 3.51 | 3.59 | 4.12 | 3.98 | 4.26 | 0.352 | 0.056 | <0.001 |
| Spain | 3.78 | 3.74 | 3.82 | 3.73 | 3.69 | 3.77 | 4.09 | 4.00 | 4.19 | 0.256 | 0.041 | <0.001 |
| Sweden | 3.98 | 3.94 | 4.02 | 3.95 | 3.92 | 3.99 | 4.44 | 4.31 | 4.58 | 0.341 | 0.062 | <0.001 |
| Switzerland | 3.52 | 3.50 | 3.55 | 3.49 | 3.47 | 3.52 | 4.20 | 4.09 | 4.31 | 0.635 | 0.052 | <0.001 |
| Turkey | 3.63 | 3.60 | 3.67 | 3.59 | 3.55 | 3.63 | 4.01 | 3.91 | 4.12 | 0.279 | 0.045 | <0.001 |
| Ukraine | 3.62 | 3.59 | 3.65 | 3.60 | 3.56 | 3.63 | 3.91 | 3.78 | 4.04 | 0.240 | 0.054 | <0.001 |
| Wales | 3.83 | 3.81 | 3.85 | 3.77 | 3.75 | 3.79 | 4.20 | 4.15 | 4.25 | 0.321 | 0.027 | <0.001 |
| Notes: SMU = social media use; LL = confidence interval lower limit; UL = confidence interval upper limit; *β* = STDY-standardized (i.e., *B*/standard deviation(Y)), controlled for gender, age, and socioeconomic status; *SE* = standard error; *p* = p-value. | | | | | | | | | | | | |

| **Table S13: Summary of sensitivity analysis** | | | | | | | | | | | |
| --- | --- | --- | --- | --- | --- | --- | --- | --- | --- | --- | --- |
|  | **Cut-off 6-9** | | | **Cut-off 5-9** | | | | **Cut-off 7-9** | | | |
|  | **Countries^1^** | ***β* average^2^** | ***β* pooled^3^** | **Countries^1^** | ***β* average^2^** | ***β* pooled^3^** | ***r*^4^** | **Countries^1^** | ***β* average^2^** | ***β* pooled^3^** | ***r* ^5^** |
| Problematic SMU and life satisfaction | 40 | -0.397 | -0.395 | 42 | -0.371 | -0.372 | 0.953 | 40 | -0.417 | -0.408 | 0.934 |
| Problematic SMU and psychosomatic complaints | 44 | 0.654 | 0.648 | 44 | 0.620 | 0.609 | 0.938 | 43 | 0.682 | 0.670 | 0.929 |
| Problematic SMU and intensity of online communication | 41 | 0.301 | 0.313 | 42 | 0.285 | 0.309 | 0.948 | 39 | 0.300 | 0.294 | 0.940 |
| Notes: SMU = social media use; *β* = STDY-effect size (i.e., *B*/standard deviation(Y)), controlled for gender, age, and socioeconomic status.  ^1^ Number of countries where a significant association was observed in the same direction as in the pooled sample.  ^2^ Average magnitude of the association computed from the effect sizes of 44 countries.  ^3^ Magnitude of the associated computed from the pooled sample.  ^4^ Correlation between the effect sizes based on a 5-9 cut-off and the effect sizes based on a 6-9 cut-off (*n* = 44 countries).  ^5^ Correlation between the effect sizes based on a 7-9 cut-off and the effect sizes based on a 6-9 cut-off (*n* = 44 countries). | | | | | | | | | | | |

**References**

[1] Van den Eijnden RJJM, Lemmens J, Valkenburg PM. The Social Media Disorder Scale: Validity and psychometric properties. Comput Human Behav 2016;61:478–87; https://doi.org/10.1016/j.chb.2016.03.038.

[2] Boer M, Stevens GWJM, Finkenauer C, et al. Validation of the Social Media Disorder Scale in Dutch adolescents: Findings from a large-scale nationally representative sample. Assessment 2021 https://doi.org/10.1177/10731911211027232.

[3] Boer M, Van den Eijnden RJJM, Boniel-Nissim M, et al. Adolescents’ intense and problematic social media use and their wellbeing in 29 countries. J Adolesc Heal 2020;66:S89–99; https://doi.org/10.1016/j.jadohealth.2020.02.014.

[4] Ledesma RD, Valero-mora P. Determining the Number of Factors to Retain in EFA. Pract Assessment, Res Eval 2007;12:1–3;.

[5] Hu LT, Bentler PM. Cutoff criteria for fit indexes in covariance structure analysis: Conventional criteria versus new alternatives. Struct Equ Model 1999;6:1–55; https://doi.org/10.1080/10705519909540118.

[6] Fabrigar LR, Wegener DT, MacCallum RC, et al. Evaluating the use of exploratory factor analysis in psychological research. Psychol Methods 1999;4:272–99; https://doi.org/10.1016/0743-9547(91)90011-L.

[7] Costello AB, Osborne JW. Best practices in exploratory factor analysis: Four recommendations for getting the most from your analysis. Pract Assessment, Res Educ 2005;10:1–9; https://doi.org/10.1.1.110.9154.

[8] Bagozzi RP, Yi Y. On the evaluation of structural equation models. J Acad Mark Sci 1988;16:74–94; https://doi.org/10.1007/BF02723327.

[9] Howard MC. A Review of Exploratory Factor Analysis Decisions and Overview of Current Practices: What We Are Doing and How Can We Improve? Int J Hum Comput Interact 2016;32:51–62; https://doi.org/10.1080/10447318.2015.1087664.
